# Plasma protein N-glycome composition associates with postprandial lipaemic response

**DOI:** 10.1186/s12916-023-02938-z

**Published:** 2023-07-03

**Authors:** Panayiotis Louca, Tamara Štambuk, Azra Frkatović-Hodžić, Ana Nogal, Massimo Mangino, Sarah E. Berry, Helena Deriš, George Hadjigeorgiou, Jonathan Wolf, Martina Vinicki, Paul W. Franks, Ana M. Valdes, Tim D. Spector, Gordan Lauc, Cristina Menni

**Affiliations:** 1 Department of Twin Research and Genetic Epidemiology, King’s College London, St Thomas’ Hospital Campus, Westminster Bridge Road, London, SE1 7EH UK; 2 Genos Glycoscience Research Laboratory, Zagreb, Croatia; 3 NIHR Biomedical Research Centre at Guy’s and St Thomas’ Foundation Trust, London, SE1 9RT UK; 4 Department of Nutritional Sciences, King’s College London, Franklin Wilkins Building, London, SE1 9NH UK; 5 ZOE Ltd, London, UK; 6 Lund University Diabetes Center, Lund University, Malmö, Sweden; 7 Department of Clinical Sciences, Lund University, Malmö, Sweden; 8 Academic Rheumatology Clinical Sciences Building, Nottingham City Hospital, University of Nottingham, Nottingham, UK; 9 University of Zagreb Faculty of Pharmacy and Biochemistry, Zagreb, Croatia

**Keywords:** Protein glycosylation, Plasma N-glycome, Postprandial glycaemia, Postprandial lipaemia, Metabolic response

## Abstract

**Background:**

A dysregulated postprandial metabolic response is a risk factor for chronic diseases, including type 2 diabetes mellitus (T2DM). The plasma protein N-glycome is implicated in both lipid metabolism and T2DM risk. Hence, we first investigate the relationship between the N-glycome and postprandial metabolism and then explore the mediatory role of the plasma N-glycome in the relationship between postprandial lipaemia and T2DM.

**Methods:**

We included 995 individuals from the ZOE-PREDICT 1 study with plasma N-glycans measured by ultra-performance liquid chromatography at fasting and triglyceride, insulin, and glucose levels measured at fasting and following a mixed-meal challenge. Linear mixed models were used to investigate the associations between plasma protein N-glycosylation and metabolic response (fasting, postprandial (*C*_max_), or change from fasting). A mediation analysis was used to further explore the relationship of the N-glycome in the prediabetes (HbA1c = 39–47 mmol/mol (5.7–6.5%))–postprandial lipaemia association.

**Results:**

We identified 36 out of 55 glycans significantly associated with postprandial triglycerides (*C*_max_ *β* ranging from -0.28 for low-branched glycans to 0.30 for GP26) after adjusting for covariates and multiple testing (*p*_adjusted_ < 0.05). N-glycome composition explained 12.6% of the variance in postprandial triglycerides not already explained by traditional risk factors. Twenty-seven glycans were also associated with postprandial glucose and 12 with postprandial insulin. Additionally, 3 of the postprandial triglyceride–associated glycans (GP9, GP11, and GP32) also correlate with prediabetes and partially mediate the relationship between prediabetes and postprandial triglycerides.

**Conclusions:**

This study provides a comprehensive overview of the interconnections between plasma protein N-glycosylation and postprandial responses, demonstrating the incremental predictive benefit of N-glycans. We also suggest a considerable proportion of the effect of prediabetes on postprandial triglycerides is mediated by some plasma N-glycans.

**Supplementary Information:**

The online version contains supplementary material available at 10.1186/s12916-023-02938-z.

## Background

The majority of the population spend most of their waking day in a postprandial state, a dynamic, non-steady state with rapid fluctuations in glucose, insulin, triglycerides, and inflammatory levels [1]. These postprandial excursions are significant indicators of overall metabolic control [1], and postprandial lipaemia and glycaemia are independent risk factors for the development of cardiovascular and other chronic diseases [2] due to their downstream effects on lipoprotein remodelling, inflammation, oxidative stress, and haemostatic remodelling [3].

Glycosylation is an essential and highly regulated posttranslational modification present on most human proteins [4]. N-glycosylation is the most common form of glycosylation and is highly responsive to genetic, epigenetic, and environmental stimuli [4]. The N-glycome integrates information on immunological (immunoglobulins), inflammatory (acute phase reactants, immunoglobulins), and metabolic (apolipoproteins, AGP) components [4]. One measure of N-glycosylation, glycoprotein acetylation (GlycA), reflects the concentration and glycosylation state of many acute phase glycoproteins, including α1-acid glycoprotein and haptoglobin [4]. Recent studies report GlycA to be a marker of inflammation [5] and found it to associate with CVD [6], type-2 diabetes mellitus (T2DM) [7], and triglyceride levels [8]. Moreover, GlycA levels have been shown to change postprandially [2] illustrating the effect diet can have on glycosylation levels. However, to date, there are no published studies investigating the correlations of the complete N-glycome and postprandial responses.

We have previously reported that the complete N-glycome is associated with T2DM, and changes in plasma protein N-glycome composition were found to predict incident T2DM up to 10-years prior to disease onset [9]. These plasma N-glycome changes are gradual and show continuous deterioration toward the clinical manifestation of diabetes [10]. Moreover, previous research suggests that individuals with T2DM are dyslipidaemic, and postprandial levels of triglycerides are an independent risk factor for atherosclerosis in individuals with T2DM [11].

Here, we aimed to explore the correlations and predictive capacity of the N-glycome and postprandial triglyceride, glucose, and insulin responses in the ZOE PREDICT-1 study [1]. Then, given the causal links between glycans and diabetes and correlations with lipid metabolism, we focus on lipaemia and further investigate the mediatory role of the N-glycome in the prediabetes–postprandial lipaemia association.

## Methods

**Fig. 1:**
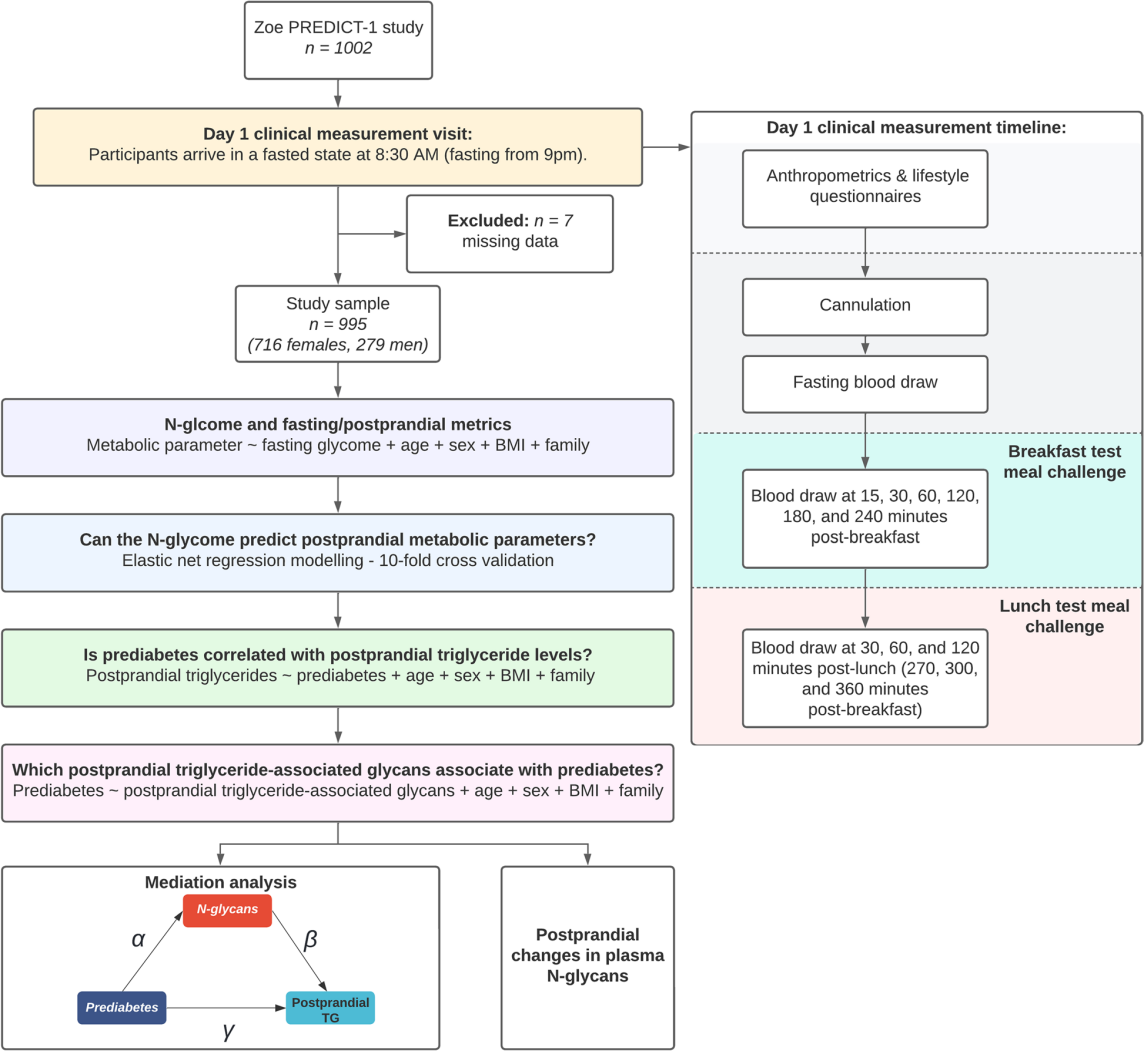
Flowchart of the study analytical pipeline and ZOE PREDICT-1 baseline clinical visit

A flowchart of the study design is presented in Fig. 1.

### Study population

The ZOE PREDICT-1 study was a single-arm nutritional intervention study—conducted between 5 June 2018 and 8 May 2019—designed to quantify and predict individual variations in postprandial triglyceride, glucose, and insulin responses to standardised meals [1]. Study participants were aged between 18 and 65 years recruited from the TwinsUK registry [12] and the general population using online advertising. Participants attended a full day clinical visit consisting of test meal challenges followed by a 13-day home-based phase, as previously described [1].

Data relevant to this analysis pertain to the day 1 baseline clinical measurement visit at St. Thomas’ Hospital, illustrated within Fig. 1 [13]; see Additional file 1: Supplementary methods. Outcome variables were blood triglyceride, glucose, and insulin measured at specific timepoints and increments following test meal challenges [1]. For each of these variables, we considered (i) the baseline fasting measures, (ii) the peak concentration (*C*_max_) (over the 6-h visit for triglycerides, and 2 h for insulin and glucose, if participants had measurements at each increment), and (iii) the magnitude of increase (delta = peak − baseline), based on our previous reports (see Berry et al. [1]). Given that the ZOE PREDICT-1 study recruited generally healthy individuals, to model the relationship with diabetes, we used impaired fasting glucose, otherwise known as prediabetes. Using HbA1c levels, participants were classified as prediabetic cases if their HbA1c was within 39–47 mmol/mol (5.7–6.5%) [14]; otherwise, they were considered as controls.

### Plasma protein N-glycan profiling

Plasma N-glycans were profiled at fasting and again 4 h after the consumption of the first metabolic challenge meal in a sub-sample of the population. Detailed description of plasma protein N-glycan profiling is included in Additional file 1: Supplementary methods. Briefly, plasma samples (*V* = 10 μl) were denatured and treated with amidase PNGase F (Promega, USA) to cleave N-glycans from plasma proteins. Released plasma protein N-glycans were labelled with a fluorescent label 2-aminobenzamide (2-AB) and subsequently purified by hydrophilic interaction liquid chromatography solid-phase extraction (HILIC-SPE) using a 0.2-μm wwPTFE filter plate (Pall Corporation, USA). Fluorescently labelled and purified N-glycans were profiled by HILIC on Acquity ultra-performance liquid chromatography (UPLC) H-Class instrument (Waters, USA). The instrument consisted of a quaternary solvent manager, a sample manager and a fluorescence detector, controlled by Empower 3 software, build 3471 (Waters, USA). For separation of plasma N-glycans, Waters BEH Glycan chromatography column was used. The resulting chromatograms were all separated in the same manner into 39 glycan peaks (GP1–GP39) (Additional file 2: Supplementary Fig. 1). The amount of glycans in each peak was expressed as a percentage of the total integrated area. A list of N-glycan structures corresponding to each glycan peak is available in Additional file 3: Supplementary table 1. Given that individual glycans share common glycosylation features, including galactosylation, fucosylation, bisecting GlcNAc, and sialylation, derived traits were constructed to represent the proportion of these features. Sixteen derived glycan traits were approximated from the proportion of directly measured glycan peaks (GP1-GP39), each of which combined the glycans with the same structural characteristics (Additional file 3: Supplementary table 2).

### Statistical analysis

All statistical analyses were computed using R statistical software, version 3.5.1 [15]. Normalisation and batch correction were performed on UPLC glycan measurements to remove experimental variation. Total area normalisation was performed to make the measurements comparable, followed by the log transformation to obtain normally distributed data. Batch correction was performed using the ComBat function in the R package ‘sva’ [16] by modelling the plate on which the sample was analysed as the batch covariate. Subsequently, the values were transformed back to the original scale.

The association of postprandial responses (fasted, peak (*C*_max_), and delta values) with each plasma glycan was tested using linear mixed models, adjusting for age, sex, body mass index (BMI), and family relatedness as a random effect (Fig. 1). The values were transformed to standard normal distribution prior to fitting by using the ‘*rntransform*’ function in the R package ‘GenABEL’ [17]. We controlled for false discovery rate using Benjamini–Hochberg procedure (*p*_adjusted_ < 0.05) [18].

We then investigated to which extent the baseline fasted plasma N-glycans could predict postprandial response (either peak (*C*_max_) or delta) using elastic net regression (R package ‘glmnet’ [19]). Several models including different sets of predictors were built for comparison:

$$1.\,traditional\,risk\,factors \left(age+sex+BMI\right)$$

$$2.\,N-glycome$$

$$3.\,traditional\,risk\,factors+N-glycome$$

$$4.\,traditional\,risk\,factors+GlycA$$

$$5.\,traditional\,risk\,factors+N-glycome+GlycA$$

Regularisation was applied to all included predictors. Prior to model training, a linear mixed model was used to account for the twin pairs (random effect) in the sample set. The residuals of the model were then centred and scaled. A 10-fold cross validation procedure was used to evaluate the performance, and the average prediction from all test folds was used to calculate *R*^2^.

Next, we assessed the relationship between prediabetes and postprandial triglycerides (*C*_max_) and which postprandial triglyceride-associated glycans also associated with prediabetes using linear mixed models adjusting for age, sex, BMI, and family. After which, we explored the mediatory role of plasma N-glycans (indirect effect) in the relationship between prediabetes and triglycerides. As outlined by Baron and Kenny [20], this multi-step process includes (step one) determining the correlation between prediabetes and triglycerides (the direct effect), (step two) prediabetes and N-glycans, and (step three) N-glycans and triglycerides (while including prediabetes). Mediation analysis was performed using the ‘*mediate*’ function in the R package ‘mediation’ (version 4.5.0) [21], adjusting for age, sex, and BMI. The significance of the indirect effect of triglycerides on prediabetes status was determined using bootstrapping procedure, where unstandardised indirect effects was computed for each of 1000 bootstrapped samples. A *p* value < 0.05 indicates a significant mediatory effect. The 95% confidence interval was determined by taking the indirect effect values at the 2.5th and 97.5th percentiles. The variance accounted for (VAF) was determined as the ratio of indirect-to-total effect $\frac{Indirect effect}{Total effect}$ and describes the proportion of the variance explained by the mediation process (the proportion of the effect of prediabetes on triglycerides that goes through N-glycans).

In a sub-analysis, we further investigated postprandial changes in plasma protein N-glycans using a linear mixed model where time was modelled as a fixed effect, while the individual ID was considered as a random effect.

## Results

We included 995 healthy adults (716 women and 279 men), including 122 twin pairs, with an average age of 45.6 ± 12 years, and a mean BMI of 25.6 ± 5 kg/m^2^ from the ZOE-PREDICT 1 study. Demographic characteristics, cardiometabolic blood biomarkers, and metrics of postprandial responses are presented in Table 1.

**Table 1:** Descriptive characteristics of the study participants (*n* = 995)

**Phenotype**	
Females (*n* (%)*)*	716 (72)
Twin pairs (*n* (%))	122 (24.5)
Age (years)	45.6 (12)
BMI (kg/m^2^)	25.6 (5.1)
*Triglycerides*	
Fasting triglyceride concentration (mmol/L)	1.1 (0.5)
Peak triglyceride concentration (*C*_max 0-6 h_) (mmol/L)	2.3 (1.2)
*Glucose*	
Fasting plasma glucose concentration (mmol/L)	5 (0.5)
Peak plasma glucose concentration (*C*_max 0-2 h_) (mmol/L)	7.3 (1.2)
*Insulin*	
Fasting insulin concentration (mU/l)	6.2 (4.3)
Peak insulin concentration (*C*_max 0-2 h_) (mU/l)	72.7 (40.7)
*Inflammation*	
Fasting GlycA (mmol/L)	1.3 (0.2)

Data presented as mean (SD) unless otherwise indicated

*Abbreviations*: *BMI* Body mass index, *GlycA* Glycoprotein acetylation, *SD* Standard deviation

### Association of plasma protein N-glycosylation with postprandial lipaemia

**Fig. 2:**
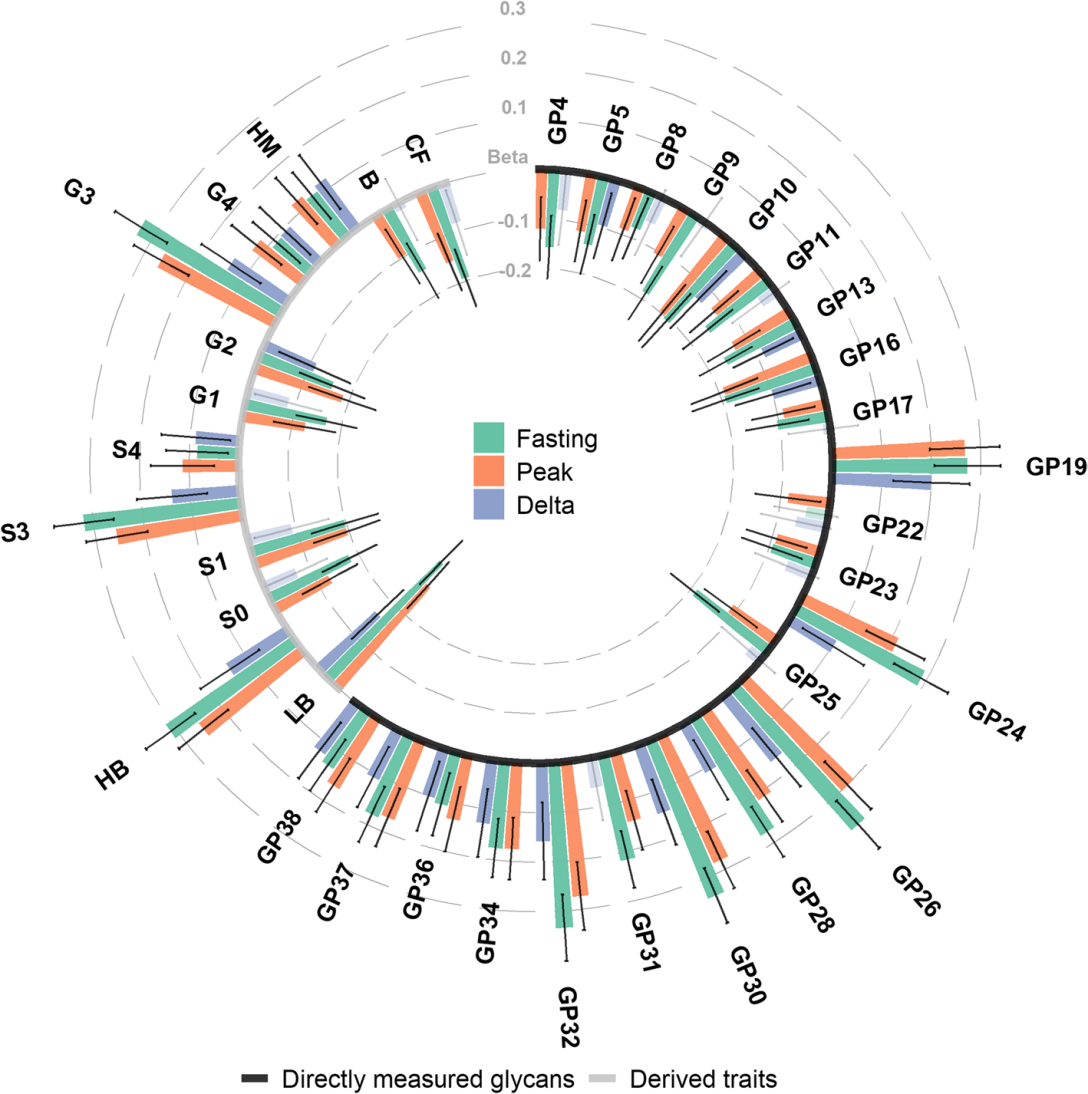
Association of plasma N-glycans with postprandial triglyceride levels. Effect size is represented in standard deviation units. Error bars represent 95% confidence intervals. AF, antennary fucosylation; B, bisecting GlcNAc; CF, core fucosylation; GP, glycan peak; G0, agalactosylation; G1, monogalactosylation; G2, digalactosylation; G3, trigalactosylation; G4, tetragalactoslation; HM, high mannose; HB, high branching; LB, low branching; S0, asialylation; S1, monosialylation; S2, disialylation; S3, trisialylation; S4, tetrasialylation. N-glycan structures corresponding to each glycan peak (GP) are listed in Supplementary table 1. Transparent bars represent non-significant associations at *p*_adjusted_ threshold < 0.05

We first examined the associations between plasma protein N-glycans and postprandial levels of triglycerides, glucose, and insulin (both *C*_max_ and change from fasting). Postprandial triglyceride response (*C*_max_) showed the strongest association with the plasma protein N-glycome. Indeed, significant associations were observed between triglyceride peak concentrations (*C*_max 0-6 h_) and 36 out of 55 glycan traits (65.5%), after adjusting for covariates and multiple testing (Additional file 3: Supplementary table 3, Fig. 2). The strongest associations were with glycan peaks GP26 (glycan structures FA3G3S2 and A3G3S2) (*β* = 0.30 (*SE* = 0.03), *p*_adjusted_ = 1.49 × 10^−15^) and GP30 (A3G3S3 and A3F1G3S3) (*β* = 0.28 (0.03), *p*_adjusted_ = 2.55 × 10^−15^), and levels of low-branched glycans (*β* =  − 0.28 (0.03), *p*_adjusted_ = 2.55 × 10^−15^) as well as trigalactosylated (G3; *β* = 0.26 (0.03), *p*_adjusted_ = 6.78 × 10^−14^), trisialylated (S3) (*β* = 0.26 (0.03), *p*_adjusted_ = 1.56 × 10^−13^), and high-branched glycans (*β* = 0.26 (0.03), *p*_adjusted_ = 2.32 × 10^−13^). Consistent, although weaker, associations were observed for both fasting and delta triglyceride concentrations (see Additional file 3: Supplementary table 3).

The associations between the of plasma protein N-glycome composition and glycaemic and insulin response were less widespread across the N-glycome and are described in the Additional file 4: Supplementary results. Briefly, 27 and 12 glycan traits were significantly associated with glucose and insulin peaks (*C*_max_), respectively, after adjusting for covariates and multiple testing (Additional file 3: Supplementary table 4, Supplementary table 5, and Additional file 2: Supplementary Fig. 2).

### Predictive capacity of plasma protein N-glycosylation on postprandial peaks (C_max_)

**Fig. 3:**
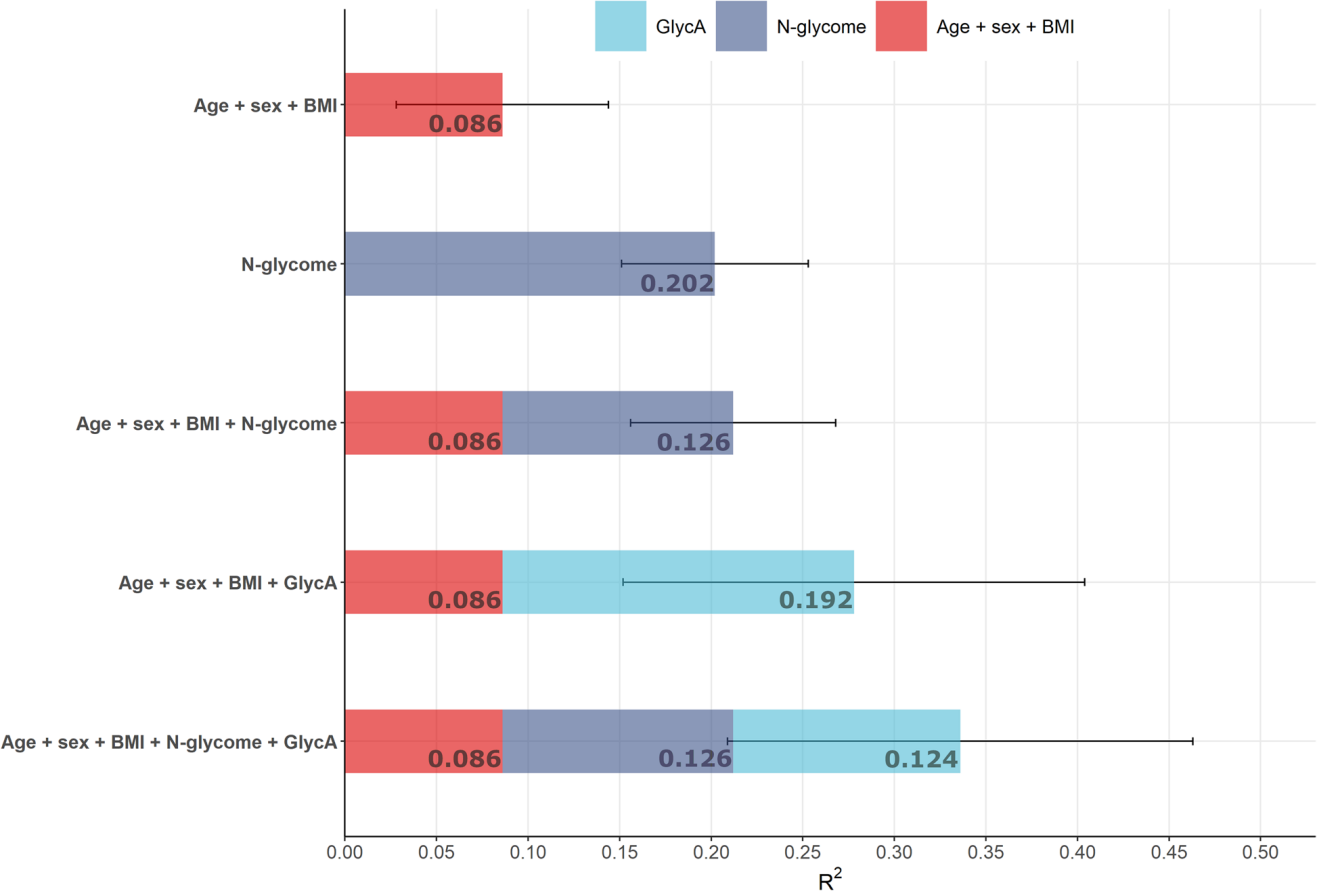
The variance explained for lipaemic response across various prediction models. Bars depict *R*^2^ in descending order, while error bars depict standard deviation. BMI, body mass index; GlycA, glycoprotein acetylation

For triglycerides, the plasma protein N-glycome explained an additional 12.6% of the variance in postprandial lipaemic peak (*C*_max_) compared to a traditional risk factor model (age, sex, and BMI). Alone the N-glycome explained 20% of peak postprandial triglyceride response, while a model combining the traditional risk factors and the N-glycome explained 21%. The further addition of GlycA to the model increases the variance explained to 33.6% (Fig. 3).

We also tested the predictive capacity of postprandial glycaemic and insulin responses using fasted protein N-glycome composition. The results of which are described in Additional file 4: Supplementary results. Briefly, traditional risk factors could predict 5% of postprandial glucose peak, while the plasma N-glycome could predict 7%, combined this accumulated to 8%, and the further addition of GlycA increased this to 9%. While for insulin, traditional risk factors predicted 10% of postprandial insulin peak, and the N-glycome alone predicted 8%, combined this equated to 11% (Additional file 2: Supplementary Fig. 3).

### Relationship between triglycerides, plasma protein N-glycosylation and prediabetic status

**Fig. 4:**
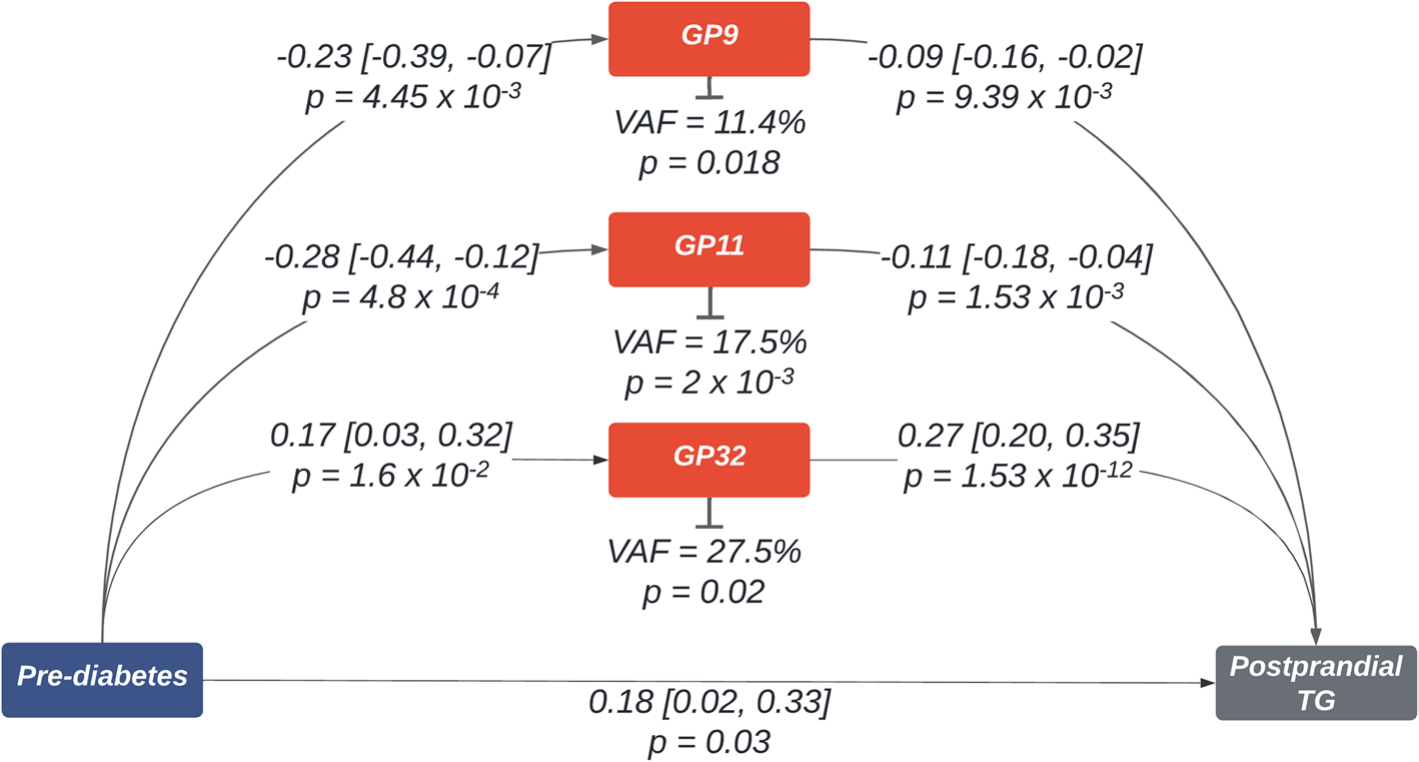
Mediation analysis diagram. Prediabetes was modelled as the exposure. Postprandial Triglyceride peak (*C*_max_) was the outcome and N-glycans associated with both exposure and outcome were modelled as mediators. Coefficients depicted are beta [95% CI]

In line with previous literature [11], while adjusting for age, sex, BMI, and family, we found that prediabetes status (HbA1c = 39–47 mmol/mol (5.7–6.5%)) was significantly associated with postprandial triglyceride concentrations (*C*_max_) (Beta [95% CI] = 0.18 [0.02, 0.33]; *p* = 2.51 × 10^−2^) (step 1). We have previously reported correlations between insulin resistance/T2DM and several plasma glycans [10]; hence, we tested the 36 glycans that were associated with peak postprandial triglycerides (*C*_max_) with prediabetes using linear mixed effect models. After adjusting for age, sex, BMI, and family, 2 glycans, GP9 and GP11, were negatively associated with prediabetes, and GP32 was positively associated (step 2). These 3 glycans were also associated with postprandial triglycerides while further adjusting for prediabetes (step 3). Following causal mediation analyses, all 3 significantly mediated the relationship between prediabetes and postprandial triglyceride response (Fig. 4).

### Postprandial alterations in plasma protein N-glycosylation

**Fig. 5:**
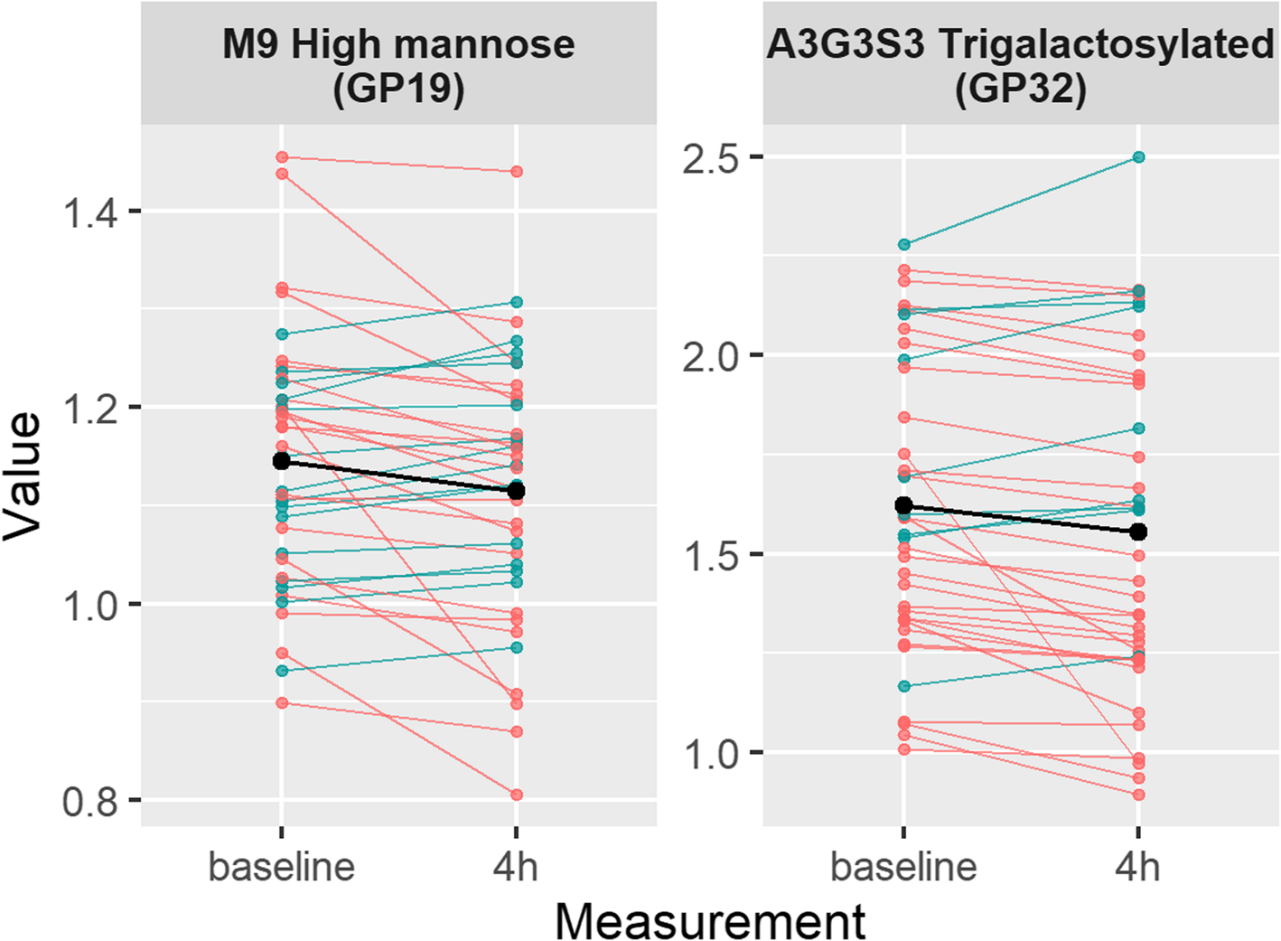
Postprandial change in relative abundance of plasma protein N-glycans GP19 and GP32. Green lines denote an increase in glycan abundance, while red lines indicate a decrease. Mean values are shown in black

Next, in a sub-analysis, we explored the postprandial changes in the plasma protein N-glycome in 38 participants that had glycan data available at 2 time points (fasted and 4 h after the consumption of the first metabolic challenge meal) using a linear-mixed model. We observed significant postprandial decreases in GP19 (M9) (*β* =  − 0.23 (standard error = 0.08), *p*_adjusted_ = 0.032) and GP32 (A3G3S3) (*β* =  − 0.22 (standard error = 0.08), *p*_adjusted_ = 0.021), while accounting for covariates and multiple testing (Fig. 5). Results were consistent when further accounting for the 2 twin pairs within this sample.

## Discussion

This is the first study to comprehensively assess the link between plasma protein N-glycosylation and postprandial metabolic responses. We find that the fasting plasma protein N-glycome is strongly associated with postprandial peak triglyceride levels, particularly glycan structures FA3G3S2 and A3G3S2, A3G3S3 and A3F1G3S3, and levels of low-branched glycans. Moreover, we find that the plasma N-glycome can explain an extra 12.6% of the variance in peak postprandial triglycerides, compared to traditional risk factors. We further showed that 3 plasma N-glycans correlated with prediabetes status and in fact these mediate between 11 and 27% of the effect of prediabetes on peak postprandial triglyceride levels. Lastly, we are the first to report that 2 plasma N-glycans, high mannose M9, and high-branched A3G3S3 decrease following a meal challenge.

The plasma protein glycans we report to be univariately associated with postprandial lipaemia are complex, highly-branched, trigalactosylated, and highly sialylated structures—FA3G3S2, A3G3S2, A3G3S3, A3F1G3S3, alongside a single high mannose glycan—M9. Consistent effects with branched, highly galactosylated and sialylated glycans were also observed for postprandial glycaemic and insulin response. This detrimental health effect of these glycan structure is consistent with previous evidence, which has reported associations with these glycan structures and increased risk of incident T2DM, poorer metabolic control, and an increase in cardiovascular events [9, 10, 22].

Interestingly, we also find postprandial changes in two of these glycan structures, GP19 (M9) and GP32 (A3G3S3). Dietary intake is a known contributor to the glycosylation of proteins, particularly higher sialylation with healthier dietary patterns [23]. But, to date, this is the first study to demonstrate acute effects. The M9 glycan (GP19) is known to predominantly originate from apolipoprotein B-100. Apolipoprotein B-100 is the core structural protein of very low-density lipoprotein (VLDL) particles [24]. Lipoproteins have been shown to be dysfunctional and of altered composition/size in T2DM [25, 26], and their apolipoprotein and remodelling protein constituents can be readily glycosylated [27]. In our data, we report a postprandial decrease in M9 reflecting reduced liver secretion of VLDL particles in response to increased postprandial insulin levels and increased influx of chylomicron particles carrying exogenous triglycerides [28]. A3G3S3 (GP32) has multiple protein sources [29], and reduced levels might reflect the recruitment of alpha-1 acid glycoprotein in response to the increased nutrient flux [30, 31]. The lack of postprandial changes in the other plasma glycan structures is likely due to the short period between the two measurements (4 h), since most of the plasma glycoproteins have a half-life ranging from 2 to 5 days (except transferrin (8–10 days) and immunoglobulin G (~ 23 days)).

We further examined, in a multivariable model, whether the fasting composition of the plasma N-glycome could predict postprandial metabolic response to standardized meals. We found that the N-glycome explained 20% of the variance in peak triglyceride concentrations, more than double that of a traditional risk factor model (age, sex, and BMI (9%)). Moreover, we highlight the incremental utility of the N-glycome and GlycA in the prediction of peak triglyceride concentrations. Where a model integrating traditional risk factors, the N-glycome, and GlycA exhibited the best overall performance, explaining more than one third (34%) of the variation. This is likely due to complementary information on inflammatory and metabolic status of an individual that the N-glycome and GlycA incorporate, which are both significant determinants of cardiometabolic risk [9, 32, 33].

Numerous studies have found strong links between insulin resistance or prediabetes on postprandial lipaemia [11, 34–36]. Importantly, we have previously shown that several glycans including GP10, GP16, GP18, GP19, GP20, and GP34 are significantly associated with insulin resistance and T2DM [10], and in this study, we report an association between prediabetes and peak postprandial triglyceride concentrations We therefore investigated if the effect of prediabetes on postprandial triglyceride levels could be mediated in-part by the three specific glycans related to prediabetes (GP9, GP11, and GP32) which are also associated with postprandial triglycerides. Indeed, all 3 plasma N-glycans were significantly mediating this relationship. This is consistent with previous research which implicate inflammation and vascular function as possible mediators of the pathological impact of postprandial lipemia, and glycosylation is known to play a significant role in these processes [6, 37, 38]. Given the correlation between prediabetes and postprandial lipaemia, and postprandial decreases in M9, a glycan linked with apolipoproteins, the mediatory role of N-glycans in the prediabetes and lipaemia association may also be due to impaired lipoprotein structure and function owing to modified glycosylation [25–27]. However, we are unable to know whether fasted N-glycans are causal in this relationship. To dissect the directionality of this relationship, studies designed specifically to measure exposure, mediator, and outcome in that specific temporal order, while accounting for all potential confounders are necessary [39].

This study identifies a significant relationship between the N-glycome, postprandial lipaemia, and prediabetes. However, despite the integral nature of glucose and insulin levels to T2DM development, our work suggests that the N-glycome is less involved in postprandial glucose and insulin responses, suggesting that the mechanisms at play are mainly inflammatory and vascular. Indeed, the relationship between insulin/glucose and prediabetes is considered direct (inadequate insulin secretion and inefficient insulin sensitivity of the liver, which fails to downregulate glucose levels). However, this finding may also be explained by the generally healthy nature of the study participants with an average peak postprandial glucose concentration of 7.33 mmol/L while glycosylation is induced by levels > 7.8–8.9 mmol/L [40].

Our study has several strengths—it used a mixed nutrient sequential test meal challenge. While studying the impact of nutrition and dietary components on risk profiles, most research has taken a very simplistic approach, examining the effect of a single determinant in isolation. However, the combined impact of multiple factors, representing a more realistic scenario should be considered. It is also the first study to thoroughly explore the link between plasma protein N-glycosylation and postprandial metabolic responses, where it revealed extensive associations between the two. It also demonstrated the value of fasting plasma N-glycome composition in predicting postprandial lipaemic response and proposed for the first time that plasma protein glycosylation may mediate the effect of glucose dysregulation on postprandial lipemia.

The current study also has several limitations—firstly, the plasma protein N-glycome was not profiled at all available time points, which disabled us from exploring the full longitudinal dynamics of glycosylation changes in respect to postprandial metabolic response. Second, as the ZOE PREDICT-1 trial participants were generally healthy, we were unable to explore direct relationships with T2DM and instead were only able to explore impaired fasting glucose (prediabetes). Third, our mediation analysis also had some limitations, including (i) a relatively low sample size for such analysis (only 237 participants with prediabetes) and (ii) no evidence of temporal relationship between exposure, mediator, and outcome, which would, again, require longitudinal glycomic data to confirm the exact triangular relationship. Fourth, the design of our study prevents any inferences of directionality or causality. Fifth, our study sample is predominantly female (72%), thus limiting the generalisability of our findings. Sixth, the scope of our study did not include functional elucidation of plausible pathways that could explain the observed changes in glycosylation. Therefore, mechanisms remain speculative and future studies should seek to explore this. Lastly, although our outcome (triglyceride response) was the response of a tightly controlled intervention (standardised meal), the potential endogenous factors that may influence lipid metabolism are vast (reviewed in [41]) and include multiple psychological factors, visceral adiposity, and sex, requiring in-depth focussed research to elucidate.

## Conclusions

This study comprehensively studies the interconnections between the plasma protein N-glycome and postprandial responses, demonstrates the incremental improvement of the N-glycome in predicting lipaemic response, and suggests a mediatory role within the relationship between prediabetes and postprandial lipaemia. Further in-depth analyses are necessary to understand causality and confounding within this relationship and the mechanistic role of specific N-glycans or glycosylation features in the relationship between and impaired fasting glucose and postprandial triglycerides.

## Supplementary Information

**Additional file 1.** Supplementary methods.

**Additional file 2: Supplementary Figure 1.** Representative HILIC-UPLC-FLR chromatogram of plasma protein N-glycome. **Supplementary Figure 2.** Association of plasma glycans with postprandial glucose and insulin levels. **Supplementary Figure 3.** The variance explained for glycaemic response and insulin response across various prediction models.

**Additional file 3: Supplementary Table 1.** List of glycan structures corresponding to every individual plasma protein glycan peak. **Supplementary Table 2.** Plasma protein derived glycan traits calculated out of 39 initial plasma glycan peaks. **Supplementary Table 3.** Associations between plasma protein N-glycome and triglycerides. **Supplementary Table 4.** Associations between plasma protein N-glycome and glucose. **Supplementary table 5.** Associations between plasma protein N-glycome and insulin.

**Additional file 4.** Supplementary results.

## Data Availability

The data used in this study are held by the department of Twin Research at King’s College London. The data can be released to bona fide researchers using our normal procedures overseen by the Wellcome Trust and its guidelines as part of our core funding (https://twinsuk.ac.uk/resources-for-researchers/access-our-data/).

## Abbreviations

2-AB: 2-Aminobenzamide
BMI: Body mass index
GlycA: Glycoprotein acetylation
GP: Glycan peak
HILIC-SPE: Hydrophilic interaction liquid chromatography solid-phase extraction
T2DM: Type-2 diabetes mellitus
UPLC: Ultra-performance liquid chromatography
VAF: Variance accounted for
VLDL: Very low-density lipoprotein
