# Supplementary material for: Plasma protein N-glycome composition associates with postprandial lipaemic response
Source: BMC Med. 2023 Jul 3;21:231. doi: 10.1186/s12916-023-02938-z (PMC10318725; doi:10.1186/s12916-023-02938-z)
Supplement: Supplementary file 1 — Additional file 1. Supplementary methods. [file 12916_2023_2938_MOESM1_ESM.docx]

**Supplementary methods**

**ZOE Personalized REsponses to DIetary Composition (PREDICT) 1 study clinical visit**

During their visit, participants arrived at 8:30 am in a fasted state (fasting from 9pm the previous night). Participants provided baseline characteristics, including age, sex, and anthropometric measurements, and were then cannulated using the antecubital vein for blood sampling. Within a tightly controlled clinical setting, participants consumed meal 1: breakfast muffins and a milkshake (890 kcal, 85.5g carbohydrate (38.4%), 52.7g fat (53.3%), 16.1g protein (7.2%), and 2.3g fibre at 0h) and meal 2: lunch muffins (502 kcal, 71.2g carbohydrate (56.7%), 22.2g fat (39.8%), 9.6g protein (7.6%) and 2.2g fibre at 4h). Participants were permitted to sip water throughout. Venous blood samples were collected at 15, 30, 60, 120, 180, 240, 300, 360 minutes post meal 1.

**Plasma protein N-glycan analysis**

***Plasma protein N-glycan enzymatic release***

Denaturation of plasma samples (10 μl) was performed using 2% SDS (Invitrogen, USA) followed by incubation at 65 °C for 10 min. Subsequently, 10 μl of 4% Igepal-CA630 (Sigma Aldrich, USA) was added to the denatured samples. N-glycans were enzymatically cleaved from plasma proteins with the addition of 1.2 U of PNGase F (Promega, USA) followed by overnight incubation at 37 °C.

***Plasma protein N-glycan labelling and HILIC-SPE clean up***

Released plasma protein N-glycans were labelled with a fluorescent label 2-aminobenzamide (2-AB). The labelling mixture consisted of 2-AB (Sigma Aldrich, USA) and 2-picoline borane (Sigma Aldrich, USA) in dimethyl sulfoxide (Sigma Aldrich, USA) and glacial acetic acid (Merck, Germany) mixture (70:30). Total volume of 25 μl of this mixture was added to each sample, followed by a 2h incubation at 65 °C. Glycan purification was performed by hydrophilic interaction liquid chromatography solid-phase extraction (HILIC-SPE) using a 0.2 μm wwPTFE filter plate (Pall Corporation, USA). Later, the samples were brought to 96% of acetonitrile (ACN; Sigma Aldrich, USA) and applied to each well of the filter plate. Loaded samples were subsequently washed 5× with 96% ACN, N-glycans were eluted with ultrapure water and stored at -20°C until further analysis.

***Plasma protein N-glycan chromatographic profiling***

Plasma protein N-glycans were profiled by HILIC on Acquity ultra performance liquid chromatography (UPLC) H-Class instrument (Waters, USA). The instrument consisted of a quaternary solvent manager (QSM), a sample manager (SM) and a fluorescence (FLR) detector, controlled by Empower 3 software, build 3471 (Waters, USA). Waters BEH Glycan chromatography column was used for N-glycan separation. The calibration of the system was performed using an external standard containing hydrolysed and 2-AB labelled glucose oligomers, which were used for the conversion of individual glycans’ retention times to glucose units (GU). Data processing was performed using a semiautomatic processing method with a traditional integration algorithm after which each chromatogram was manually corrected to maintain the same intervals of integration for all the samples. The chromatograms were all separated in the same manner into 39 glycan peaks (GP1–GP39) (**Supplementary figure 1**). The amount of glycans in each peak was expressed as a percentage of the total integrated area. List of N-glycan structures corresponding to each glycan peak is available in **Supplementary table 1**. Common glycosylation features, such as sialylation, galactosylation, fucosylation, bisection, and degree of glycan branching were described by 16 derived glycan traits, that were calculated using 39 initial plasma glycan peaks (**Supplementary table 2).**
