# Supplementary material for: Plasma protein N-glycome composition associates with postprandial lipaemic response
Source: BMC Med. 2023 Jul 3;21:231. doi: 10.1186/s12916-023-02938-z (PMC10318725; doi:10.1186/s12916-023-02938-z)
Supplement: Supplementary file 2 — Additional file 2: Supplementary Figure 1. Representative HILIC-UPLC-FLR chromatogram of plasma protein N-glycome. Supplementary Figure 2. Association of plasma glycans with postprandial glucose and insulin levels. Supplementary Figure 3. The variance explained for glycaemic response and insulin response across various prediction models. [file 12916_2023_2938_MOESM2_ESM.docx]

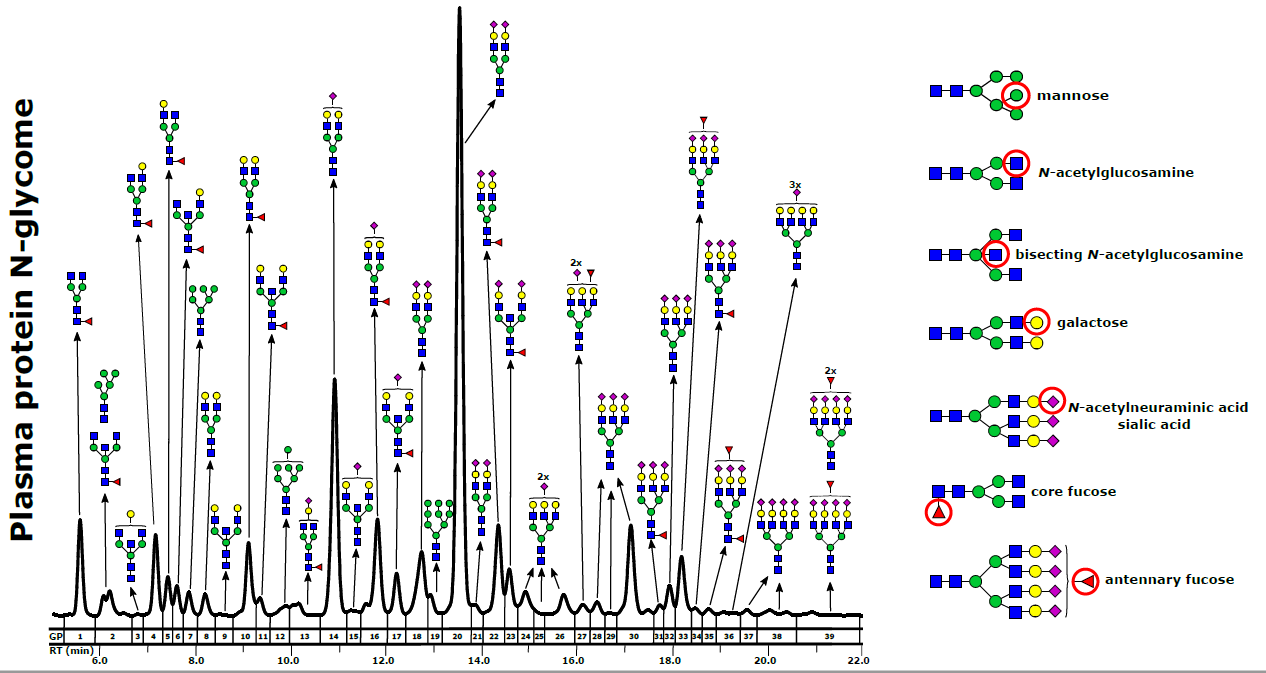


**Supplementary figure 1.** **Representative HILIC-UPLC-FLR chromatogram of plasma protein N-glycome, with graphic representation of the most abundant glycan structure corresponding to each glycan peak**.


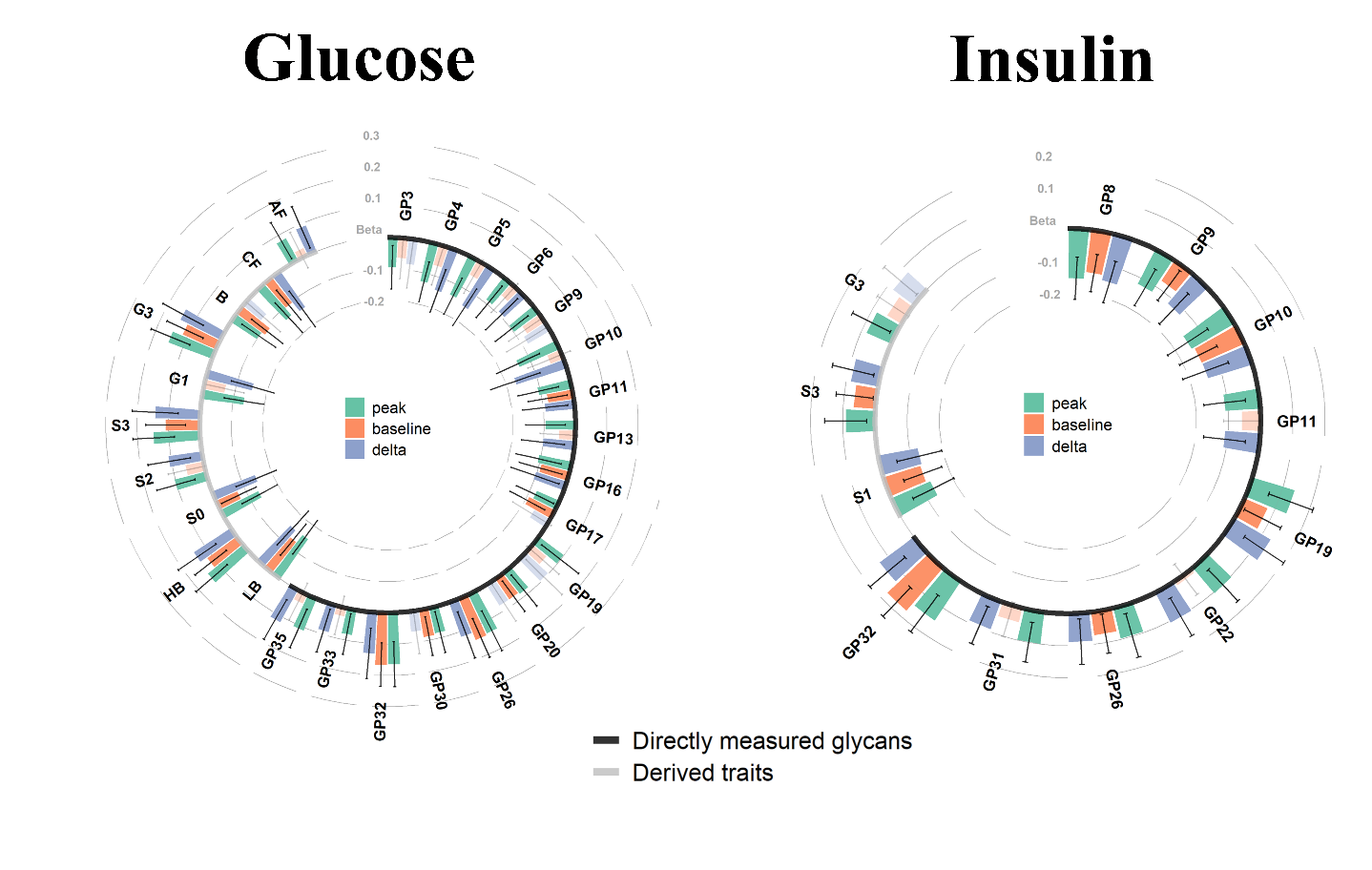


**Supplementary figure 2**: **Association of plasma glycans with postprandial glucose and insulin levels.** Effect size is represented in standard deviation (SD) units. Error bars represent 95% confidence intervals. Abbreviations: AF - antennary fucosylation; B - bisecting GlcNAc; CF - core fucosylation; GP - glycan peak; G0 - agalactosylation; G1 - monogalactosylation; G2 - digalactosylation; G3 - trigalactosylation; G4 - tetragalactoslation; HM - high mannose; HB - high branching; LB - low branching; S0 - asialylation; S1 - monosialylation; S2 - disialylation; S3 - trisialylation; S4 – tetrasialylation. N-glycan structures corresponding to each glycan peak (GP) are listed in Supplementary Table 1 Transparent bars represent non-significant associations at p_adjusted_ threshold < 0.05.


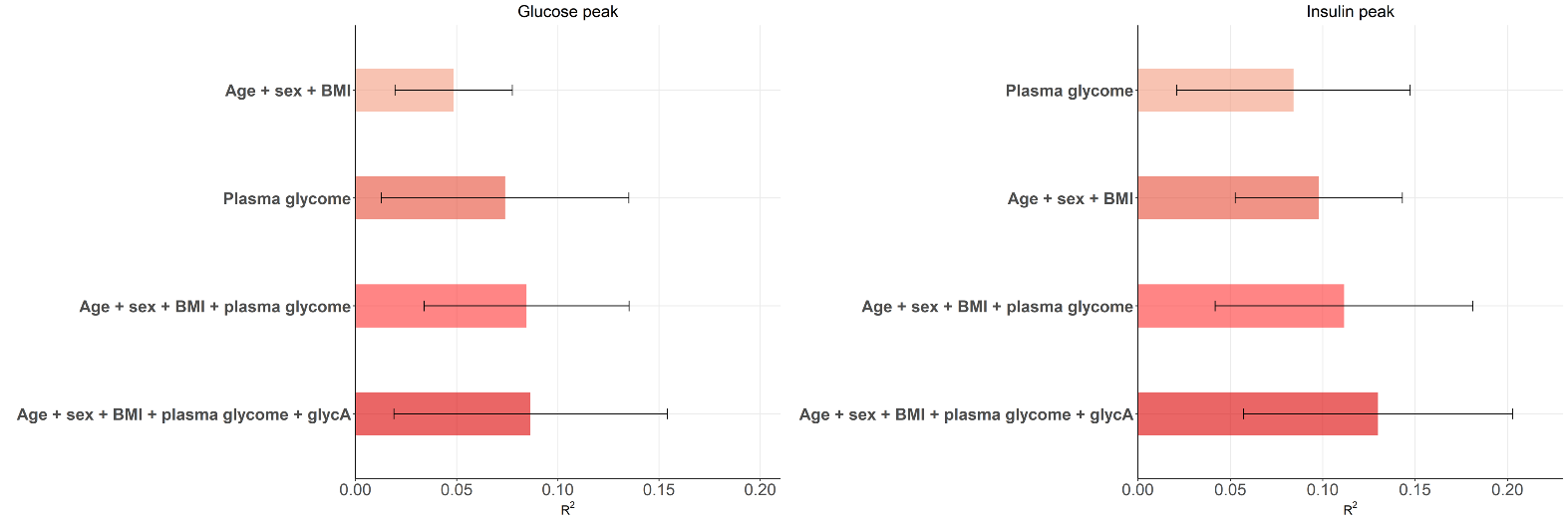


**Supplementary figure 3** **The variance explained for glycaemic response (left) and insulin response (right) across various prediction models.**
Bars depict R^2^ in descending order, while error bars depict standard deviation. Abbreviations: BMI – body mass index; GlycA – glycoprotein acetylation.
