# Supplementary material for: Plasma protein N-glycome composition associates with postprandial lipaemic response
Source: BMC Med. 2023 Jul 3;21:231. doi: 10.1186/s12916-023-02938-z (PMC10318725; doi:10.1186/s12916-023-02938-z)
Supplement: Supplementary file 3 — Additional file 3: Supplementary Table 1. List of glycan structures corresponding to every individual plasma protein glycan peak. Supplementary Table 2. Plasma protein derived glycan traits calculated out of 39 initial plasma glycan peaks. Supplementary Table 3. Associations between plasma protein N-glycome and triglycerides. Supplementary Table 4. Associations between plasma protein N-glycome and glucose. Supplementary table 5. Associations between plasma protein N-glycome and insulin. [file 12916_2023_2938_MOESM3_ESM.docx]

**Supplementary table 1. List of glycan structures corresponding to every individual plasma protein glycan peak (GP).**

| Glycan peak | Glycan structure | Description | Formula |
| --- | --- | --- | --- |
| GP1 | FA2 | core fucosylated, biantennary | GP1 / GP * 100 |
| GP2 | FA2B;  M5 | core fucosylated, biantennary with bisecting GlcNAc;  high mannose | GP2 / GP * 100 |
| GP3 | A2BG1 | monogalactosylated, biantennary with bisecting GlcNAc | GP3 / GP * 100 |
| GP4 | FA2[6]G1 | core fucosylated and monogalactosylated, biantennary | GP4 / GP * 100 |
| GP5 | FA2[3]G1 | core fucosylated and monogalactosylated, biantennary | GP5 / GP * 100 |
| GP6 | FA2[6]BG1 | core fucosylated and monogalactosylated, biantennary with bisecting GlcNAc | GP6 / GP * 100 |
| GP7 | M6;  FA2[3]BG1 | high mannose;  core fucosylated and monogalactosylated, biantennary with bisecting GlcNAc | GP7 / GP * 100 |
| GP8 | A2G2 | digalactosylated, biantennary | GP8 / GP * 100 |
| GP9 | A2BG2 | digalactosylated, biantennary with bisecting GlcNAc | GP9 / GP * 100 |
| GP10 | FA2G2 | core fucosylated, digalactosylated, biantennary | GP10 / GP * 100 |
| GP11 | FA2BG2 | core fucosylated, digalactosylated, biantennary with bisecting GlcNAc | GP11 / GP * 100 |
| GP12 | M7;  A2G2S1;  A2BG1S1 | high mannose;  digalactosylated, monosialylated, biantennary;  monogalactosylated, monosialylated, biantennary with bisecting GlcNAc | GP12 / GP * 100 |
| GP13 | FA2G1S1;  FA2BG1S1 | core fucosylated, monogalactosylated and monosialylated biantennary;  core fucosylated, monogalactosylated and monosialylated biantennary with bisecting GlcNAc | GP13 / GP * 100 |
| GP14 | A2G2S1 | digalactosylated and monosialylated biantennary | GP14 / GP * 100 |
| GP15 | A2BG2S1 | digalactosylated and monosialylated biantennary with bisecting GlcNAc | GP15 / GP * 100 |
| GP16 | FA2G2S1 | core fucosylated, digalactosylated and monosialylated biantennary | GP16 / GP * 100 |
| GP17 | FA2BG2S1 | core fucosylated, digalactosylated and monosialylated biantennary with bisecting GlcNAc | GP17/ GP * 100 |
| GP18 | A2G2S2;  FA2G2S2 | digalactosylated and disialylated biantennary;  core fucosylated, digalactosylated and disialylated biantennary | GP18 / GP * 100 |
| GP19 | M9 | high mannose | GP19 / GP * 100 |
| GP20 | A2G2S2 | digalactosylated and disialylated biantennary | GP20 / GP * 100 |
| GP21 | A2G2S2;  A3G3S1;  FA2G2S2;  A2BG2S2 | digalactosylated and disialylated biantennary;  trigalactosylated and monosialylated triantennary;  core fucosylated, digalactosylated and disialylated biantennary;  digalactosylated and disialylated biantennary with bisecting GlcNAc | GP21 / GP * 100 |
| GP22 | FA2G2S2 | core fucosylated, digalactosylated and disialylated biantennary | GP22 / GP * 100 |
| GP23 | FA2BG2S2 | core fucosylated, digalactosylated and disialylated biantennary with bisecting GlcNAc | GP23 / GP * 100 |
| GP24 | A3G3S2;  A3F1G3S1 | trigalactosylated and disialylated triantennary;  antennary fucosylated, trigalactosylated and monosialylated triantennary | GP24 / GP * 100 |
| GP25 | A3G3S2;  FA2F1G2S2;  A3F1G3S2 | trigalactosylated and disialylated triantennary;  core fucosylated, antennary fucosylated, digalactosylated and disialylated biantennary;  antennary fucosylated, trigalactosylated and disialylated triantennary | GP25 / GP * 100 |
| GP26 | A3G3S2;  FA3G3S2 | trigalactosylated and disialylated triantennary;  core fucosylated, trigalactosylated and disialylated triantennary | GP26 / GP * 100 |
| GP27 | A3F1G3S2;  A3G3S3 | antennary fucosylated, trigalactosylated and disialylated triantennary;  trigalactosylated and trisialylated triantennary | GP27 / GP * 100 |
| GP28 | A3G3S3;  A3F1G3S2 | trigalactosylated and trisialylated triantennary;  antennary fucosylated, trigalactosylated and disialylated triantennary | GP28 / GP * 100 |
| GP29 | A3G3S3;  A3F1G3S2 | trigalactosylated and trisialylated triantennary;  antennary fucosylated, trigalactosylated and disialylated triantennary | GP29 / GP * 100 |
| GP30 | A3G3S3;  A3F1G3S3 | trigalactosylated and trisialylated triantennary;  antennary fucosylated, trigalactosylated and trisialylated triantennary | GP30 / GP * 100 |
| GP31 | FA3G3S3;  A3G3S3 | core fucosylated, trigalactosylated and trisialylated triantennary;  trigalactosylated and trisialylated triantennary | GP31 / GP * 100 |
| GP32 | A3G3S3 | trigalactosylated and trisialylated triantennary | GP32 / GP * 100 |
| GP33 | A3F1G3S3 | antennary fucosylated, trigalactosylated and trisialylated triantennary | GP33 / GP * 100 |
| GP34 | FA3G3S3;  A4G4S3 | core fucosylated, trigalactosylated and trisialylated triantennary;  tetragalactosylated and trisialylated tetraantennary | GP34 / GP * 100 |
| GP35 | FA3F1G3S3;  A4F1G4S3 | core fucosylated, antennary fucosylated, trigalactosylated and trisialylated triantennary;  antennary fucosylated, tetragalactosylated and trisialylated tetraantennary | GP35 / GP * 100 |
| GP36 | A4G4S3;  A4F1G4S3 | tetragalactosylated and trisialylated tetraantennary;  antennary fucosylated, tetragalactosylated and trisialylated tetraantennary | GP36 / GP * 100 |
| GP37 | A4G4S4;  A4F1G4S3 | tetragalactosylated and tetrasialylated tetraantennary;  antennary fucosylated, tetragalactosylated and trisialylated tetraantennary | GP37 / GP * 100 |
| GP38 | A4G4S4;  A4F1G4S4;  A4F1G4S3 | tetragalactosylated and tetrasialylated tetraantennary;  antennary fucosylated, tetragalactosylated and tetrasialylated tetraantennary;  antennary fucosylated, tetragalactosylated and trisialylated tetraantennary | GP38 / GP * 100 |
| GP39 | A4F1G4S4;  A4F2G4S4 | antennary fucosylated, tetragalactosylated and tetrasialylated tetraantennary;  antennary difucosylated, tetragalactosylated and tetrasialylated tetraantennary; | GP39 / GP * 100 |

*Structure abbreviations – all N-glycans have two core N-acetylglucosamines (GlcNAcs); F at the start of the abbreviation indicates a core-fucose α1,6-linked to the inner GlcNAc; Mx, number (x) of mannose on core GlcNAcs; Ax, number of antenna (GlcNAc) on trimannosyl core; A2, biantennary with both GlcNAcs as β1,2-linked; A3, triantennary with a GlcNAc linked β1,2 to both mannose and the third GlcNAc linked β1,4 to the α1,3 linked mannose; A4, GlcNAcs linked as A3 with additional GlcNAc β1,6 linked to α1,6 mannose; B, bisecting GlcNAc linked β1,4 to β1,3 mannose; G(x), number (x) of β1,4 linked galactose on antenna; F(x), number (x) of fucose linked α1,3 to antenna GlcNAc; S(x), number (x) of sialic acids linked to galactose.*

**Supplementary table 2.** **Plasma protein derived glycan traits calculated out of 39 initial plasma glycan peaks (GPs).**

| Structural feature | Formula |
| --- | --- |
| Low branching (mono- and biantennary glycans) (LB) | GP1+GP2+GP3+GP4+GP5+GP6+GP8+GP9+GP10+GP11+0.5xGP12+GP13+GP14+GP15+GP16+GP17+GP18+GP20+GP21+GP22+GP23 |
| High branching (tri- and tetraantennary glycans) (HB) | GP24+GP25+GP26+GP27+GP28+GP29+GP30+GP31+GP32+GP33+GP34+ GP35+GP36+GP37+GP38+GP39 |
| Agalactosylation (G0) | GP1+GP2 |
| Monogalactosylation (G1) | GP3+GP4+GP5+GP6+GP13 |
| Digalactosylation (G2) | GP8+GP9+GP10+GP11+0.5xGP12+GP14+GP15+GP16+GP17+GP18+GP20+GP21+GP22+GP23 |
| Trigalactosylation (G3) | GP24+GP25+GP26+GP27+GP28+GP29+GP30+GP31+GP32+GP33+GP34+ GP35 |
| Tetragalactosylation (G4) | GP36+GP37+GP38+GP39 |
| Neutral glycans (S0) | GP1+GP2+GP3+GP4+GP5+GP6+GP8+GP9+GP10+GP11 |
| Monosialylation (S1) | 0.5xGP12+GP13+GP14+GP15+GP16+GP17 |
| Disialylation (S2) | GP18+GP20+GP21+GP22+GP23+GP24+GP25+GP26+GP27 |
| Trisialylation (S3) | GP28+GP29+GP30+GP31+GP32+GP33+GP34+GP35+GP36 |
| Tetrasialylation (S4) | GP37+GP38+GP39 |
| Incidence of bisecting GlcNAc (B) | GP2+GP3+GP6+GP9+GP11+GP15+GP17+GP23 |
| Antennary fucosylation (AF) | GP27+GP33+GP35+GP39 |
| Core fucosylation (CF) | GP1+GP2+GP4+GP5+GP6+GP10+GP11+GP13+GP16+GP17+GP22+GP23+GP31+GP34+GP35 |
| High mannose glycans (HM) | GP7+0.5xGP12+GP19 |

**Supplementary table 3.** **Associations between plasma protein N-glycome and triglycerides.** Associations are expressed as β coefficients, resulting from linear-mixed models. Age, sex, and BMI were included as covariates, while false discovery rate was controlled using Benjamini-Hochberg method. (n = 866)

|  | | Fasting triglyceride concentration | | | Triglyceride peak concentration (C_max 0-6h_) | | | | Triglyceride delta concentration | | |  |
| --- | --- | --- | --- | --- | --- | --- | --- | --- | --- | --- | --- | --- |
| Glycan | **Effect (β)** | | **SE** | **Adjusted p-value** | | **Effect (β)** | **SE** | **Adjusted p-value** | **Effect (β)** | **SE** | **Adjusted p-value** | |
| GP1 | -0.093 | | 0.035 | 2.14E-02 | | -0.027 | 0.036 | 5.73E-01 | -0.015 | 0.039 | 7.85E-01 | |
| GP2 | -0.094 | | 0.035 | 2.16E-02 | | -0.002 | 0.037 | 9.67E-01 | 0.054 | 0.040 | 2.81E-01 | |
| GP3 | -0.122 | | 0.035 | 1.70E-03 | | -0.077 | 0.035 | 7.31E-02 | -0.016 | 0.038 | 7.76E-01 | |
| GP4 | -0.157 | | 0.032 | 1.09E-05 | | -0.121 | 0.033 | 1.19E-03 | -0.080 | 0.036 | 7.49E-02 | |
| GP5 | -0.140 | | 0.032 | 5.53E-05 | | -0.117 | 0.032 | 1.35E-03 | -0.096 | 0.035 | 2.37E-02 | |
| GP6 | -0.120 | | 0.032 | 9.32E-04 | | -0.075 | 0.033 | 6.02E-02 | -0.017 | 0.036 | 7.58E-01 | |
| GP7 | -0.009 | | 0.032 | 8.36E-01 | | 0.026 | 0.033 | 5.60E-01 | 0.062 | 0.036 | 1.64E-01 | |
| GP8 | -0.079 | | 0.034 | 4.35E-02 | | -0.090 | 0.034 | 2.62E-02 | -0.056 | 0.037 | 2.15E-01 | |
| GP9 | -0.186 | | 0.032 | 1.06E-07 | | -0.107 | 0.033 | 5.52E-03 | -0.012 | 0.037 | 8.08E-01 | |
| GP10 | -0.205 | | 0.037 | 4.30E-07 | | -0.198 | 0.038 | 2.38E-06 | -0.130 | 0.041 | 9.36E-03 | |
| GP11 | -0.156 | | 0.032 | 1.21E-05 | | -0.123 | 0.033 | 9.72E-04 | -0.038 | 0.036 | 4.20E-01 | |
| GP12 | 0.016 | | 0.033 | 6.95E-01 | | 0.023 | 0.034 | 6.08E-01 | 0.025 | 0.037 | 6.40E-01 | |
| GP13 | -0.161 | | 0.031 | 1.45E-06 | | -0.132 | 0.031 | 1.89E-04 | -0.087 | 0.034 | 4.20E-02 | |
| GP14 | 0.024 | | 0.032 | 5.32E-01 | | -0.006 | 0.032 | 8.78E-01 | -0.020 | 0.035 | 7.09E-01 | |
| GP15 | -0.115 | | 0.033 | 1.77E-03 | | -0.055 | 0.033 | 1.73E-01 | 0.019 | 0.036 | 7.19E-01 | |
| GP16 | -0.193 | | 0.036 | 1.01E-06 | | -0.190 | 0.036 | 3.32E-06 | -0.099 | 0.040 | 4.62E-02 | |
| GP17 | -0.104 | | 0.033 | 4.66E-03 | | -0.088 | 0.033 | 2.31E-02 | -0.014 | 0.036 | 7.78E-01 | |
| GP18 | 0.045 | | 0.035 | 2.84E-01 | | 0.024 | 0.035 | 6.03E-01 | 0.011 | 0.038 | 8.26E-01 | |
| GP19 | 0.273 | | 0.034 | 6.24E-14 | | 0.268 | 0.036 | 6.72E-12 | 0.201 | 0.039 | 6.70E-05 | |
| GP20 | 0.055 | | 0.031 | 1.41E-01 | | 0.040 | 0.032 | 3.16E-01 | 0.009 | 0.035 | 8.34E-01 | |
| GP21 | -0.002 | | 0.028 | 9.50E-01 | | 0.005 | 0.030 | 8.95E-01 | 0.032 | 0.032 | 4.50E-01 | |
| GP22 | -0.046 | | 0.033 | 2.42E-01 | | -0.086 | 0.034 | 3.31E-02 | -0.062 | 0.037 | 1.68E-01 | |
| GP23 | -0.095 | | 0.033 | 1.15E-02 | | -0.092 | 0.032 | 1.59E-02 | -0.054 | 0.035 | 2.13E-01 | |
| GP24 | 0.289 | | 0.032 | 1.47E-17 | | 0.212 | 0.034 | 8.40E-09 | 0.103 | 0.037 | 2.17E-02 | |
| GP25 | -0.189 | | 0.032 | 3.89E-08 | | -0.112 | 0.033 | 3.18E-03 | -0.032 | 0.036 | 5.07E-01 | |
| GP26 | 0.374 | | 0.033 | 1.05E-25 | | 0.302 | 0.034 | 1.49E-15 | 0.163 | 0.039 | 8.03E-04 | |
| GP27 | -0.108 | | 0.032 | 3.05E-03 | | -0.064 | 0.033 | 1.10E-01 | -0.030 | 0.036 | 5.29E-01 | |
| GP28 | 0.274 | | 0.031 | 3.78E-17 | | 0.211 | 0.032 | 2.53E-09 | 0.108 | 0.035 | 1.17E-02 | |
| GP29 | -0.090 | | 0.032 | 1.57E-02 | | -0.067 | 0.033 | 9.63E-02 | -0.038 | 0.036 | 4.19E-01 | |
| GP30 | 0.341 | | 0.031 | 3.93E-25 | | 0.279 | 0.032 | 2.55E-15 | 0.145 | 0.036 | 1.07E-03 | |
| GP31 | 0.216 | | 0.030 | 2.18E-11 | | 0.143 | 0.032 | 6.63E-05 | 0.060 | 0.035 | 1.62E-01 | |
| GP32 | 0.334 | | 0.035 | 9.29E-20 | | 0.273 | 0.035 | 5.93E-13 | 0.158 | 0.040 | 1.30E-03 | |
| GP33 | -0.065 | | 0.033 | 8.77E-02 | | -0.027 | 0.034 | 5.64E-01 | -0.009 | 0.036 | 8.45E-01 | |
| GP34 | 0.176 | | 0.031 | 1.27E-07 | | 0.174 | 0.032 | 1.09E-06 | 0.128 | 0.036 | 2.34E-03 | |
| GP35 | -0.038 | | 0.034 | 3.56E-01 | | -0.019 | 0.034 | 6.70E-01 | -0.015 | 0.037 | 7.76E-01 | |
| GP36 | 0.109 | | 0.033 | 3.68E-03 | | 0.133 | 0.034 | 5.91E-04 | 0.101 | 0.038 | 2.93E-02 | |
| GP37 | 0.177 | | 0.031 | 2.22E-07 | | 0.169 | 0.032 | 2.60E-06 | 0.108 | 0.035 | 1.09E-02 | |
| GP38 | 0.136 | | 0.032 | 1.21E-04 | | 0.163 | 0.033 | 7.85E-06 | 0.121 | 0.036 | 5.01E-03 | |
| GP39 | -0.023 | | 0.032 | 5.47E-01 | | 0.021 | 0.033 | 6.33E-01 | 0.038 | 0.036 | 4.22E-01 | |
| LB | -0.328 | | 0.031 | 1.61E-22 | | -0.276 | 0.032 | 2.55E-15 | -0.163 | 0.037 | 7.60E-04 | |
| HB | 0.311 | | 0.031 | 1.19E-20 | | 0.255 | 0.032 | 2.32E-13 | 0.142 | 0.036 | 1.43E-03 | |
| S0 | -0.181 | | 0.031 | 9.90E-08 | | -0.126 | 0.032 | 5.16E-04 | -0.071 | 0.035 | 1.03E-01 | |
| S1 | -0.198 | | 0.036 | 3.86E-07 | | -0.197 | 0.036 | 9.38E-07 | -0.091 | 0.040 | 6.19E-02 | |
| S2 | 0.106 | | 0.031 | 2.35E-03 | | 0.063 | 0.032 | 1.01E-01 | 0.018 | 0.034 | 7.19E-01 | |
| S3 | 0.319 | | 0.031 | 6.90E-22 | | 0.257 | 0.032 | 1.56E-13 | 0.137 | 0.036 | 1.61E-03 | |
| S4 | 0.084 | | 0.032 | 2.21E-02 | | 0.113 | 0.033 | 2.39E-03 | 0.089 | 0.036 | 4.44E-02 | |
| G0 | -0.102 | | 0.035 | 1.17E-02 | | -0.025 | 0.036 | 6.03E-01 | 0.000 | 0.039 | 9.97E-01 | |
| G1 | -0.168 | | 0.032 | 1.06E-06 | | -0.127 | 0.032 | 4.94E-04 | -0.084 | 0.035 | 5.40E-02 | |
| G2 | -0.159 | | 0.036 | 5.87E-05 | | -0.187 | 0.036 | 4.32E-06 | -0.112 | 0.040 | 2.22E-02 | |
| G3 | 0.332 | | 0.031 | 3.78E-23 | | 0.263 | 0.032 | 6.78E-14 | 0.138 | 0.037 | 1.58E-03 | |
| G4 | 0.091 | | 0.032 | 1.38E-02 | | 0.119 | 0.033 | 1.38E-03 | 0.094 | 0.036 | 3.65E-02 | |
| HM | 0.109 | | 0.031 | 1.70E-03 | | 0.121 | 0.032 | 7.91E-04 | 0.120 | 0.035 | 3.97E-03 | |
| B | -0.144 | | 0.032 | 5.53E-05 | | -0.098 | 0.033 | 1.03E-02 | -0.015 | 0.036 | 7.76E-01 | |
| CF | -0.195 | | 0.031 | 1.00E-08 | | -0.153 | 0.032 | 1.82E-05 | -0.083 | 0.035 | 5.40E-02 | |
| AF | -0.068 | | 0.033 | 7.51E-02 | | -0.028 | 0.034 | 5.50E-01 | -0.008 | 0.036 | 8.69E-01 | |

**Supplementary table 4. Associations between plasma protein N-glycome and glucose.** Associations are expressed as β coefficients, resulting from linear-mixed models. Age, sex, and BMI were included as covariates, while false discovery rate was controlled using Benjamini-Hochberg method. (n = 903)

|  | Fasting glucose concentration | | | Glucose peak concentration (C_max 0-2h_) | | | | Glucose delta concentration | | |
| --- | --- | --- | --- | --- | --- | --- | --- | --- | --- | --- |
| Glycan | **Effect (β)** | **SE** | **Adjusted p-value** | **Effect (β)** | **SE** | **Adjusted p-value** | **Effect (β)** | | **SE** | **Adjusted p-value** |
| GP1 | -0.052 | 0.035 | 2.03E-01 | -0.072 | 0.037 | 1.03E-01 | -0.077 | | 0.040 | 1.15E-01 |
| GP2 | -0.056 | 0.035 | 1.79E-01 | -0.046 | 0.037 | 3.31E-01 | -0.016 | | 0.040 | 7.78E-01 |
| GP3 | -0.064 | 0.034 | 1.10E-01 | -0.095 | 0.036 | 2.31E-02 | -0.081 | | 0.039 | 9.22E-02 |
| GP4 | -0.068 | 0.033 | 7.51E-02 | -0.128 | 0.034 | 7.91E-04 | -0.142 | | 0.037 | 1.58E-03 |
| GP5 | -0.052 | 0.031 | 1.64E-01 | -0.138 | 0.033 | 2.20E-04 | -0.150 | | 0.035 | 8.03E-04 |
| GP6 | -0.049 | 0.032 | 1.98E-01 | -0.090 | 0.034 | 2.31E-02 | -0.092 | | 0.037 | 4.22E-02 |
| GP7 | -0.036 | 0.032 | 3.56E-01 | -0.027 | 0.033 | 5.50E-01 | -0.004 | | 0.037 | 9.37E-01 |
| GP8 | -0.028 | 0.033 | 4.99E-01 | -0.027 | 0.035 | 5.73E-01 | 0.008 | | 0.038 | 8.69E-01 |
| GP9 | -0.067 | 0.032 | 7.51E-02 | -0.098 | 0.034 | 1.32E-02 | -0.081 | | 0.037 | 7.62E-02 |
| GP10 | -0.044 | 0.037 | 3.36E-01 | -0.140 | 0.039 | 1.62E-03 | -0.169 | | 0.043 | 1.30E-03 |
| GP11 | -0.082 | 0.032 | 2.79E-02 | -0.107 | 0.034 | 6.14E-03 | -0.094 | | 0.037 | 4.17E-02 |
| GP12 | -0.025 | 0.033 | 5.24E-01 | 0.001 | 0.034 | 9.84E-01 | 0.036 | | 0.037 | 4.70E-01 |
| GP13 | -0.052 | 0.031 | 1.57E-01 | -0.096 | 0.032 | 1.17E-02 | -0.101 | | 0.035 | 1.93E-02 |
| GP14 | 0.044 | 0.031 | 2.32E-01 | 0.057 | 0.033 | 1.57E-01 | 0.078 | | 0.036 | 7.49E-02 |
| GP15 | -0.063 | 0.032 | 9.63E-02 | -0.056 | 0.034 | 1.73E-01 | -0.028 | | 0.037 | 5.81E-01 |
| GP16 | -0.093 | 0.036 | 2.73E-02 | -0.107 | 0.038 | 1.64E-02 | -0.103 | | 0.042 | 4.44E-02 |
| GP17 | -0.093 | 0.032 | 1.22E-02 | -0.080 | 0.034 | 4.80E-02 | -0.059 | | 0.037 | 1.96E-01 |
| GP18 | -0.013 | 0.034 | 7.68E-01 | 0.034 | 0.036 | 4.85E-01 | 0.064 | | 0.039 | 1.82E-01 |
| GP19 | 0.058 | 0.035 | 1.64E-01 | 0.098 | 0.038 | 2.97E-02 | 0.097 | | 0.041 | 5.40E-02 |
| GP20 | 0.079 | 0.031 | 2.73E-02 | 0.085 | 0.033 | 2.90E-02 | 0.079 | | 0.036 | 7.49E-02 |
| GP21 | -0.021 | 0.028 | 5.32E-01 | -0.004 | 0.031 | 9.14E-01 | -0.001 | | 0.033 | 9.92E-01 |
| GP22 | -0.056 | 0.033 | 1.55E-01 | 0.004 | 0.035 | 9.28E-01 | 0.016 | | 0.038 | 7.76E-01 |
| GP23 | -0.118 | 0.032 | 1.02E-03 | -0.068 | 0.033 | 8.63E-02 | -0.045 | | 0.036 | 3.34E-01 |
| GP24 | 0.077 | 0.033 | 4.45E-02 | 0.069 | 0.035 | 1.01E-01 | 0.062 | | 0.038 | 1.81E-01 |
| GP25 | 0.032 | 0.032 | 4.14E-01 | 0.066 | 0.034 | 1.04E-01 | 0.041 | | 0.037 | 3.94E-01 |
| GP26 | 0.143 | 0.035 | 1.90E-04 | 0.137 | 0.036 | 8.97E-04 | 0.116 | | 0.040 | 1.76E-02 |
| GP27 | 0.017 | 0.032 | 6.76E-01 | 0.063 | 0.034 | 1.18E-01 | 0.076 | | 0.036 | 9.22E-02 |
| GP28 | 0.045 | 0.032 | 2.29E-01 | 0.067 | 0.034 | 1.01E-01 | 0.063 | | 0.037 | 1.64E-01 |
| GP29 | -0.023 | 0.032 | 5.47E-01 | 0.012 | 0.034 | 7.90E-01 | 0.024 | | 0.037 | 6.52E-01 |
| GP30 | 0.087 | 0.032 | 1.93E-02 | 0.083 | 0.034 | 4.41E-02 | 0.065 | | 0.037 | 1.60E-01 |
| GP31 | 0.047 | 0.031 | 1.96E-01 | 0.054 | 0.033 | 1.73E-01 | 0.055 | | 0.036 | 2.13E-01 |
| GP32 | 0.166 | 0.036 | 2.10E-05 | 0.165 | 0.037 | 8.00E-05 | 0.132 | | 0.041 | 7.96E-03 |
| GP33 | 0.027 | 0.032 | 4.97E-01 | 0.081 | 0.034 | 4.68E-02 | 0.088 | | 0.037 | 4.96E-02 |
| GP34 | 0.060 | 0.031 | 9.63E-02 | 0.063 | 0.033 | 1.16E-01 | 0.055 | | 0.036 | 2.17E-01 |
| GP35 | 0.031 | 0.033 | 4.38E-01 | 0.110 | 0.034 | 5.73E-03 | 0.118 | | 0.037 | 9.15E-03 |
| GP36 | 0.105 | 0.033 | 5.16E-03 | 0.073 | 0.035 | 8.12E-02 | 0.063 | | 0.038 | 1.81E-01 |
| GP37 | 0.039 | 0.032 | 3.04E-01 | 0.023 | 0.034 | 6.02E-01 | 0.014 | | 0.036 | 7.78E-01 |
| GP38 | 0.081 | 0.032 | 2.86E-02 | 0.073 | 0.034 | 7.31E-02 | 0.061 | | 0.037 | 1.80E-01 |
| GP39 | 0.031 | 0.032 | 4.28E-01 | 0.071 | 0.034 | 7.79E-02 | 0.081 | | 0.037 | 7.49E-02 |
| LB | -0.125 | 0.033 | 6.67E-04 | -0.155 | 0.034 | 5.78E-05 | -0.153 | | 0.038 | 1.07E-03 |
| HB | 0.116 | 0.033 | 1.67E-03 | 0.145 | 0.034 | 1.65E-04 | 0.140 | | 0.038 | 1.65E-03 |
| S0 | -0.075 | 0.031 | 4.07E-02 | -0.135 | 0.033 | 2.98E-04 | -0.147 | | 0.036 | 1.03E-03 |
| S1 | -0.082 | 0.036 | 4.73E-02 | -0.079 | 0.037 | 7.74E-02 | -0.049 | | 0.041 | 3.45E-01 |
| S2 | 0.058 | 0.031 | 1.08E-01 | 0.103 | 0.033 | 6.14E-03 | 0.107 | | 0.035 | 1.17E-02 |
| S3 | 0.109 | 0.033 | 2.96E-03 | 0.149 | 0.034 | 1.16E-04 | 0.143 | | 0.037 | 1.58E-03 |
| S4 | 0.057 | 0.032 | 1.32E-01 | 0.071 | 0.033 | 7.79E-02 | 0.071 | | 0.037 | 1.18E-01 |
| G0 | -0.058 | 0.035 | 1.64E-01 | -0.072 | 0.037 | 1.05E-01 | -0.071 | | 0.040 | 1.56E-01 |
| G1 | -0.068 | 0.032 | 6.92E-02 | -0.135 | 0.033 | 3.07E-04 | -0.152 | | 0.036 | 8.03E-04 |
| G2 | -0.035 | 0.036 | 4.34E-01 | -0.023 | 0.037 | 6.41E-01 | -0.007 | | 0.041 | 8.93E-01 |
| G3 | 0.115 | 0.033 | 1.70E-03 | 0.149 | 0.034 | 1.23E-04 | 0.143 | | 0.038 | 1.58E-03 |
| G4 | 0.068 | 0.032 | 7.11E-02 | 0.073 | 0.034 | 7.31E-02 | 0.071 | | 0.037 | 1.20E-01 |
| HM | -0.007 | 0.031 | 8.59E-01 | 0.024 | 0.033 | 5.85E-01 | 0.057 | | 0.036 | 1.99E-01 |
| B | -0.113 | 0.032 | 1.87E-03 | -0.099 | 0.033 | 1.09E-02 | -0.074 | | 0.037 | 1.04E-01 |
| CF | -0.103 | 0.031 | 3.48E-03 | -0.134 | 0.033 | 3.16E-04 | -0.140 | | 0.036 | 1.43E-03 |
| AF | 0.026 | 0.032 | 5.02E-01 | 0.080 | 0.034 | 4.83E-02 | 0.090 | | 0.037 | 4.62E-02 |

**Supplementary table 5** **Associations between plasma protein N-glycome and insulin.** Associations are expressed as β coefficients, resulting from linear-mixed models. Age, sex, and BMI were included as covariates, while false discovery rate was controlled using Benjamini-Hochberg method (n = 902).

|  | Fasting insulin concentration | | | Insulin peak concentration (C_max 0-2h_) | | | | | Insulin delta concentration | | |
| --- | --- | --- | --- | --- | --- | --- | --- | --- | --- | --- | --- |
| Glycan | **Effect (β)** | **SE** | **Adjusted p-value** | | **Effect (β)** | **SE** | **Adjusted p-value** | **Effect (β)** | | **SE** | **Adjusted p-value** |
| GP1 | 0.082 | 0.031 | 2.21E-02 | | 0.052 | 0.036 | 2.39E-01 | 0.049 | | 0.036 | 2.81E-01 |
| GP2 | -0.008 | 0.032 | 8.57E-01 | | -0.033 | 0.037 | 5.14E-01 | -0.035 | | 0.037 | 4.71E-01 |
| GP3 | -0.034 | 0.031 | 3.63E-01 | | -0.057 | 0.035 | 1.78E-01 | -0.060 | | 0.035 | 1.68E-01 |
| GP4 | -0.047 | 0.029 | 1.69E-01 | | -0.052 | 0.033 | 1.98E-01 | -0.046 | | 0.034 | 2.67E-01 |
| GP5 | -0.006 | 0.029 | 8.59E-01 | | -0.038 | 0.033 | 3.65E-01 | -0.035 | | 0.033 | 4.06E-01 |
| GP6 | -0.025 | 0.029 | 4.98E-01 | | -0.060 | 0.033 | 1.34E-01 | -0.062 | | 0.033 | 1.35E-01 |
| GP7 | -0.006 | 0.029 | 8.59E-01 | | -0.017 | 0.033 | 6.92E-01 | -0.018 | | 0.033 | 7.10E-01 |
| GP8 | -0.133 | 0.030 | 5.99E-05 | | -0.155 | 0.034 | 5.78E-05 | -0.148 | | 0.034 | 8.03E-04 |
| GP9 | -0.074 | 0.030 | 2.86E-02 | | -0.120 | 0.033 | 1.47E-03 | -0.120 | | 0.033 | 2.62E-03 |
| GP10 | -0.146 | 0.033 | 7.22E-05 | | -0.154 | 0.038 | 3.93E-04 | -0.144 | | 0.039 | 1.65E-03 |
| GP11 | -0.057 | 0.029 | 9.62E-02 | | -0.109 | 0.033 | 4.39E-03 | -0.110 | | 0.033 | 6.44E-03 |
| GP12 | -0.067 | 0.030 | 5.36E-02 | | -0.060 | 0.033 | 1.40E-01 | -0.057 | | 0.034 | 1.68E-01 |
| GP13 | -0.025 | 0.028 | 4.76E-01 | | -0.037 | 0.032 | 3.60E-01 | -0.035 | | 0.032 | 4.06E-01 |
| GP14 | -0.043 | 0.029 | 2.04E-01 | | -0.047 | 0.032 | 2.39E-01 | -0.042 | | 0.033 | 3.01E-01 |
| GP15 | -0.026 | 0.029 | 4.76E-01 | | -0.072 | 0.033 | 7.38E-02 | -0.072 | | 0.034 | 8.33E-02 |
| GP16 | -0.109 | 0.033 | 2.96E-03 | | -0.086 | 0.037 | 5.41E-02 | -0.080 | | 0.038 | 8.78E-02 |
| GP17 | -0.024 | 0.029 | 5.02E-01 | | -0.058 | 0.033 | 1.54E-01 | -0.059 | | 0.033 | 1.56E-01 |
| GP18 | -0.140 | 0.031 | 3.47E-05 | | -0.081 | 0.035 | 5.90E-02 | -0.069 | | 0.036 | 1.20E-01 |
| GP19 | 0.078 | 0.032 | 3.84E-02 | | 0.138 | 0.037 | 1.13E-03 | 0.130 | | 0.038 | 3.66E-03 |
| GP20 | 0.061 | 0.029 | 6.93E-02 | | 0.027 | 0.032 | 5.44E-01 | 0.023 | | 0.033 | 6.25E-01 |
| GP21 | -0.029 | 0.026 | 3.62E-01 | | -0.021 | 0.030 | 6.01E-01 | -0.019 | | 0.030 | 6.52E-01 |
| GP22 | 0.015 | 0.030 | 6.95E-01 | | 0.104 | 0.034 | 9.06E-03 | 0.105 | | 0.034 | 1.17E-02 |
| GP23 | 0.003 | 0.029 | 9.34E-01 | | 0.014 | 0.033 | 7.45E-01 | 0.012 | | 0.033 | 7.95E-01 |
| GP24 | -0.049 | 0.030 | 1.64E-01 | | 0.002 | 0.034 | 9.60E-01 | 0.005 | | 0.035 | 8.97E-01 |
| GP25 | -0.064 | 0.029 | 6.24E-02 | | -0.056 | 0.033 | 1.67E-01 | -0.051 | | 0.033 | 2.13E-01 |
| GP26 | 0.073 | 0.032 | 4.73E-02 | | 0.097 | 0.036 | 2.24E-02 | 0.089 | | 0.036 | 4.62E-02 |
| GP27 | -0.002 | 0.029 | 9.56E-01 | | 0.004 | 0.033 | 9.23E-01 | 0.010 | | 0.033 | 8.26E-01 |
| GP28 | -0.039 | 0.029 | 2.59E-01 | | 0.028 | 0.033 | 5.44E-01 | 0.030 | | 0.034 | 5.00E-01 |
| GP29 | -0.100 | 0.029 | 2.46E-03 | | -0.048 | 0.034 | 2.46E-01 | -0.040 | | 0.034 | 3.60E-01 |
| GP30 | 0.044 | 0.030 | 2.08E-01 | | 0.065 | 0.034 | 1.07E-01 | 0.060 | | 0.034 | 1.58E-01 |
| GP31 | 0.045 | 0.028 | 1.79E-01 | | 0.100 | 0.032 | 7.08E-03 | 0.092 | | 0.032 | 1.93E-02 |
| GP32 | 0.175 | 0.032 | 4.20E-07 | | 0.147 | 0.036 | 3.35E-04 | 0.132 | | 0.037 | 2.34E-03 |
| GP33 | 0.017 | 0.029 | 6.45E-01 | | 0.023 | 0.033 | 6.03E-01 | 0.028 | | 0.034 | 5.33E-01 |
| GP34 | 0.014 | 0.029 | 6.95E-01 | | 0.046 | 0.032 | 2.50E-01 | 0.039 | | 0.033 | 3.45E-01 |
| GP35 | 0.019 | 0.030 | 5.93E-01 | | 0.061 | 0.034 | 1.40E-01 | 0.063 | | 0.034 | 1.41E-01 |
| GP36 | -0.013 | 0.030 | 7.19E-01 | | -0.036 | 0.034 | 4.20E-01 | -0.034 | | 0.034 | 4.50E-01 |
| GP37 | -0.017 | 0.029 | 6.41E-01 | | -0.017 | 0.033 | 7.00E-01 | -0.017 | | 0.033 | 7.22E-01 |
| GP38 | 0.029 | 0.029 | 4.24E-01 | | 0.000 | 0.033 | 9.98E-01 | -0.002 | | 0.033 | 9.63E-01 |
| GP39 | 0.006 | 0.029 | 8.65E-01 | | -0.013 | 0.033 | 7.55E-01 | -0.010 | | 0.033 | 8.26E-01 |
| LB | -0.048 | 0.030 | 1.69E-01 | | -0.070 | 0.034 | 8.53E-02 | -0.066 | | 0.034 | 1.18E-01 |
| HB | 0.046 | 0.030 | 1.86E-01 | | 0.070 | 0.033 | 8.48E-02 | 0.067 | | 0.034 | 1.11E-01 |
| S0 | -0.022 | 0.029 | 5.30E-01 | | -0.047 | 0.032 | 2.39E-01 | -0.045 | | 0.033 | 2.68E-01 |
| S1 | -0.120 | 0.032 | 8.08E-04 | | -0.133 | 0.036 | 1.30E-03 | -0.125 | | 0.037 | 4.02E-03 |
| S2 | 0.036 | 0.028 | 2.84E-01 | | 0.060 | 0.032 | 1.17E-01 | 0.058 | | 0.032 | 1.47E-01 |
| S3 | 0.068 | 0.030 | 4.98E-02 | | 0.091 | 0.033 | 2.16E-02 | 0.086 | | 0.034 | 4.00E-02 |
| S4 | 0.010 | 0.029 | 7.79E-01 | | -0.011 | 0.033 | 7.90E-01 | -0.011 | | 0.033 | 8.08E-01 |
| G0 | 0.065 | 0.032 | 7.85E-02 | | 0.037 | 0.037 | 4.50E-01 | 0.033 | | 0.037 | 4.93E-01 |
| G1 | -0.034 | 0.029 | 3.23E-01 | | -0.051 | 0.033 | 2.00E-01 | -0.047 | | 0.033 | 2.43E-01 |
| G2 | -0.082 | 0.032 | 2.73E-02 | | -0.081 | 0.037 | 6.70E-02 | -0.077 | | 0.037 | 9.22E-02 |
| G3 | 0.051 | 0.030 | 1.55E-01 | | 0.083 | 0.034 | 3.77E-02 | 0.080 | | 0.034 | 5.61E-02 |
| G4 | 0.006 | 0.029 | 8.59E-01 | | -0.016 | 0.033 | 7.06E-01 | -0.015 | | 0.033 | 7.63E-01 |
| HM | -0.001 | 0.028 | 9.66E-01 | | 0.013 | 0.032 | 7.45E-01 | 0.011 | | 0.032 | 8.08E-01 |
| B | -0.024 | 0.029 | 5.02E-01 | | -0.050 | 0.033 | 2.12E-01 | -0.052 | | 0.033 | 2.00E-01 |
| CF | -0.028 | 0.029 | 4.35E-01 | | -0.028 | 0.032 | 5.36E-01 | -0.026 | | 0.033 | 5.53E-01 |
| AF | 0.013 | 0.029 | 7.24E-01 | | 0.017 | 0.033 | 7.06E-01 | 0.021 | | 0.034 | 6.57E-01 |
