# Supplementary material for: Plasma protein N-glycome composition associates with postprandial lipaemic response
Source: BMC Med. 2023 Jul 3;21:231. doi: 10.1186/s12916-023-02938-z (PMC10318725; doi:10.1186/s12916-023-02938-z)
Supplement: Supplementary file 4 — Additional file 4. Supplementary results. [file 12916_2023_2938_MOESM4_ESM.docx]

**Supplementary results**

***Association of plasma protein N-glycosylation with postprandial glycaemic and insulin response***

Glucose peak concentrations (C_max 0-2h_) were significantly associated 27 plasma glycan traits, after including age, sex and BMI as covariates, and controlling for false discovery rate using Benjamini-Hochberg method (**Supplementary table 4**, **Supplementary figure 2**). Among these, the strongest associations show glycan peak GP32 (A3G3S3; β=0.17 (0.04), p_adjusted_= 8.00 x 10^-5^), low branching (LB; β=-0.16 (0.03), p_adjusted_=5.78 x10^-5^), trisialyation (S3; β=0.15 (0.03), p_adjusted_=1.16 x10^-4^), trigalactosylation (G3; β=0.15 (0.03), p_adjusted_=1.23 x10^-4^) and high branching (HB; β=0.15 (0.03), p_adjusted_=1.65 x10^-4^). Results were consistent for fasting and delta glucose values as well (**Supplementary table 4**).

Peak insulin concentrations exhibited the smallest number of significant associations with plasma N-glycome, showing 12 significant correlations, after including age, sex and BMI as covariates, and controlling for false discovery rate using Benjamini-Hochberg method (**Supplementary table 5, Supplementary figure 2**). The strongest associations were observed with glycan peaks GP8 (A2G2; β=-0.16 (0.03), p_adjusted_=5.78 x 10^-5^), GP32 (A3G3S3; β=0.15 (0.04), p_adjusted_=3.35 x 10^-4^) and GP10 (FA2G2; β=-0.15 (0.04), p_adjusted_=3.93 x 10^-4^), as well as with monosialylation (S1; β=-0.13 (0.04), p_adjusted_=1.30 x 10^-3^), trisialylation (S3; β=0.09 (0.03), p_adjusted_=2.16 x 10^-2^) and trigalactosylation (G3; β=0.08 (0.03), p_adjusted_=3.77 x 10^-2^). The associations were also consistent for fasting and delta insulin concentrations (**Supplementary table 5**).

***Prediction of postprandial lipaemic response using baseline plasma protein N-glycome composition***

In case of the postprandial glycaemic and insulin response, plasma protein N-glycome (alone or combined with age, sex, and BMI) showed the best performance in predicting peak glucose or insulin concentrations. However, the proportion of peak glucose or insulin level variance explained by baseline plasma N-glycome composition is relatively small compared to that of triglycerides (R^2^=0.07 and 0.08, respectively) (**Supplementary Figure 3**).
